# Embryonic Zebrafish as a Model for Investigating the Interaction between Environmental Pollutants and Neurodegenerative Disorders

**DOI:** 10.3390/biomedicines12071559

**Published:** 2024-07-13

**Authors:** Ji-Hang Yin, Katharine A. Horzmann

**Affiliations:** Department of Pathobiology, College of Veterinary Medicine, Auburn University, Auburn, AL 36849, USA; jzy0089@auburn.edu

**Keywords:** apoptosis, early life exposure, environmental pollutants, neurodegenerative disorders, neurotransmission, neurotoxicity, oxidative stress, zebrafish

## Abstract

Environmental pollutants have been linked to neurotoxicity and are proposed to contribute to neurodegenerative disorders. The zebrafish model provides a high-throughput platform for large-scale chemical screening and toxicity assessment and is widely accepted as an important animal model for the investigation of neurodegenerative disorders. Although recent studies explore the roles of environmental pollutants in neurodegenerative disorders in zebrafish models, current knowledge of the mechanisms of environmentally induced neurodegenerative disorders is relatively complex and overlapping. This review primarily discusses utilizing embryonic zebrafish as the model to investigate environmental pollutants-related neurodegenerative disease. We also review current applicable approaches and important biomarkers to unravel the underlying mechanism of environmentally related neurodegenerative disorders. We found embryonic zebrafish to be a powerful tool that provides a platform for evaluating neurotoxicity triggered by environmentally relevant concentrations of neurotoxic compounds. Additionally, using variable approaches to assess neurotoxicity in the embryonic zebrafish allows researchers to have insights into the complex interaction between environmental pollutants and neurodegenerative disorders and, ultimately, an understanding of the underlying mechanisms related to environmental toxicants.

## 1. Introduction

Neurodegenerative disorders are a major public health concern, with an estimate of over 50 million of people affected worldwide [1,2,3]. Aging has been considered the primary contributory risk factor to neurodegenerative disorders such as Alzheimer’s disease (AD), Parkinson’s disease (PD), and anxiety-like disorders [4,5]; however, recent studies suggest other potential causes or contributors, such as gender, genetic mutations, stress, and environmental pollutants, could increase the incidence of the disease [6,7]. Among potential causes, environmental pollutants are largely incriminated with a correlation to neurodegenerative disorders’ development [8]. The progressive production of synthetic chemical products for industrial applications and the rapid population growth have resulted in innumerable environmental chemical wastes [9]. To date, the Toxic Substances Control Act (TSCA) chemical substances inventory lists 70,000 existing chemicals in commerce [10]. Up to 30% of used chemicals may be potentially neurotoxic and play a role in neurodevelopmental diseases and disabilities [11]. The recognition of environmental toxicant-related neurologic disorders generates a high demand for establishing new approaches to assess the neurotoxic effects of environmental chemicals [11].

Since the 1950s, mammalian laboratory animals, such as rodents and rabbits, have served as the standard experimental animals for assessing toxicity in the USA [12,13]. The Organization for Economic Cooperation and Development (OECD) primarily uses rodents to evaluate the toxic characteristics of test chemicals in a variety of toxicity studies [14,15]. However, traditional methods using rodents, rabbits, and non-human primates remain costly and time-consuming [16]. Driven by the concepts of 3Rs (reduction, replacement, and refinement), European legislation regulates the use of laboratory animals for experimental and scientific purposes in European Council Directive 86/609/EEC and 2010/63/EU [17,18]. Registration, Evaluation, Authorisation, and Restriction of Chemicals (REACH) under the European Union requires that tests on vertebral animals be minimized [19]. Moreover, thousands of new chemicals are introduced to the market yearly and may, potentially, be dispersed into the environment, highlighting the importance of employing a high-throughput-based system for neurotoxicity assessment [20]. Although in vitro-cell-based assays generally follow the concept of high-throughput screening, they provide limited information on the principles of absorption, distribution, metabolism, excretion, and toxicity (ADME-Tox) [21]. In vivo models, on the other hand, largely mimic human physiology systems and provide relevant results; however, they are often not suitable for high-throughput platforms [22]. Due to the current regulations and animal welfare considerations, fish appear to be a good alternative candidate for large-scale screening in neurotoxicity studies [23]. To date, test guidelines concerning the use of fish in toxicity testing has been released by OECD including: the fish acute toxicity test (OECD test guideline 203), the fish short-term toxicity on embryos and sac-fry stages (OECD 212), the early life stage toxicity test (OECD 210), the juvenile fish growth test (OECD 215), and the bioaccumulation in fish (OECD 305) [24,25,26,27].

Several fish species are recommended for assessing toxicity study under OECD guidelines due to their availability, easy maintenance, and historical use [24]. Zebrafish are recognized as a well-established biomedical model bridging the gap between in vitro cell-based and in vivo mammalian platforms [28]. The US Environmental Protection Agency (US EPA) indicates zebrafish are valuable animals for studying developmental neurotoxicity [29,30]. The zebrafish is a powerful neurotoxicologic fish model in pharmaceutical and chemical compound screening [31]. Studies have demonstrated that zebrafish-based neurotoxic responses can predict chemical hazards in human health and ecological hazard assessments [31,32]. Using zebrafish embryos as alternatives in toxicity tests has been advocated in many studies, which proved that zebrafish embryos provided equivalent sensitivity to juveniles in the acute fish toxicity test (AFT) [33,34]. The concept of adopting zebrafish embryos in toxicity test studies is widely accepted. Following the German Federal Environment Agency’s submission and two validation studies, OECD in 2013 approved the test guideline “Fish embryo acute toxicity test” (OECD 236), particularly on zebrafish embryos, to determine the acute or lethal toxicity of test chemicals [33].

**Figure 1:**
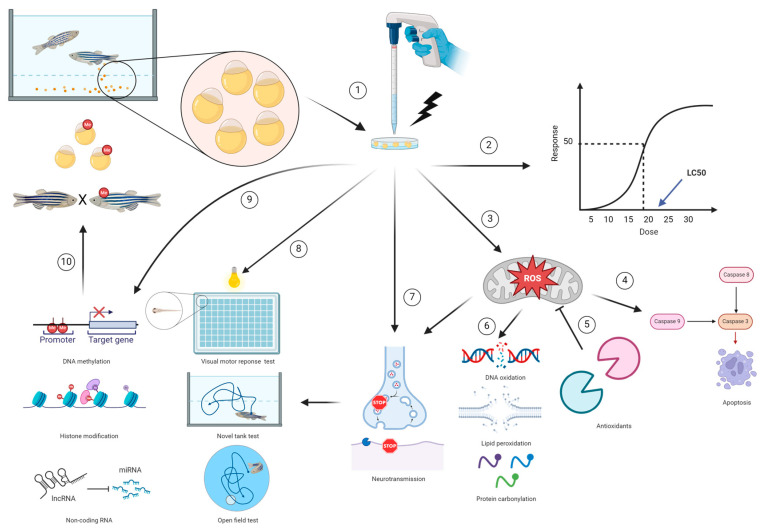
Approaches to assess neurotoxicity induced by environmentally relevant concentrations of pollutants in the zebrafish model. ① Fertilized embryonic zebrafish are collected for neurotoxicity assessment and the embryos are dosed with environmentally relevant concentrations of chemicals for assessing neurotoxicity. ② The LC50 reflects the acute toxicity of the chemicals. ③ The exposure of embryonic zebrafish to the test chemicals induces mitochondrial dysfunction and oxidative stress, which directly (chemicals) or indirectly (ROS) triggers ④ apoptosis cascades and ⑦ improper neurotransmitter release. Detection of ⑤ antioxidants and ⑥ the levels of DNA oxidation, lipid peroxidation, and protein carbonylation are common methodologies to evaluate ROS. ④ Caspases 3, 8, and 9 are frequently used biomarkers for detecting apoptosis. ⑧ Neurobehavioral tests are sensitive assays to evaluate neurotoxicity in the zebrafish model. The visual motor response test, novel tank test, and open field test are neurobehavioral assays commonly applied to neurotoxicity studies. ⑨ DNA methylation, histone modification, and non-coding RNA are parameters used to investigate the epigenetics mechanism triggered by test chemicals, ⑩ which can pass to the embryos through transgenerational epigenetic inheritance.

Recently, studies focusing on environmental pollutant-induced neurodegenerative disorders in zebrafish models have increased in number [35]. This review article is mainly focused on neurodegeneration in the zebrafish model after an early life stage environmental exposure. In addition, we investigate the pathways associated with neurodegenerative disorders and emphasize the methodologies currently discussed in zebrafish models (Figure 1).

## 2. The Zebrafish Model as a High-Throughput Platform in Neurologic Studies

The zebrafish model is a well-established platform for research studies in neurologic fields, such as neurotoxicology, neurodevelopmental disorders, neurodegenerative diseases, and drug discovery [36,37,38]. Zebrafish have a considerable anatomical and physiological resemblance to mammalian counterparts [39,40]. The zebrafish genome is fully sequenced and is reported to share 70–80% identity at the nucleotide level with the human genome and 80–90% similarity in amino acid in functional domains [41,42]. The zebrafish brain shows structural and functional similarities in neuroanatomy and neurolocalization to mammals [43]. Although the zebrafish brain lacks the amygdala, hippocampus, and substantia nigra, it has been proposed that other regions of the brain contain cells that serve similar functions [44,45].

Zebrafish are small, inexpensive, easily manipulated, and have high fecundity with a short generation time [46]. Fertilization occurs externally and zebrafish embryos are transparent during early development [47]. These features make zebrafish a suitable platform for neurodevelopmental and neurotoxicity studies through in vivo, high-throughput screening systems [31,36]. Zebrafish have a distinct, well characterized development of the central nervous system with the zebrafish brain developing early at the beginning of gastrulation around 6 h post fertilization (hpf) [48,49]. The neural signaling system, including the catecholaminergic, gamma-aminobutyric acid (GABA)-ergic, and glutamatergic systems, develops between 18 and 32 hpf [44]. The brain forms distinct regions and is divided into the forebrain (diencephalon, telencephalon), midbrain, hindbrain, and spinal cord by 24 hpf [50,51]. Primary neurons interconnected by axonal tracts occur at 21–27 hpf, and embryos start to respond to touch stimuli [52]. At 48 hpf, the zebrafish brain ventricle forms [53]. A functional blood–brain barrier presents at 3 days post fertilization (dpf) [54,55]. Glial cells, including oligodendrocytes, Schwann cells, and astrocytes, are found in zebrafish larvae at 4 dpf [56]. This rapid neurogenesis allows advanced studies to be performed in the early stages of zebrafish development. In contrast to mammals, zebrafish complete embryogenesis at 3 dpf and have most discrete organs developed at 5 dpf [57,58]. European animal welfare legislation (Directive 2010/63/EU) defines “protected vertebrae” as independently feeding larval stages [18]. Zebrafish embryos predominantly feed on yolk until 5 dpf and, therefore, are not protected by European animal welfare legislation and are not considered as independently feeding animals, which allows researchers to take advantage of this exposure window for neurotoxicity studies, such as neurobehavior [59], neurodevelopment [59], neurological drug screening [60], and receptor function [61].

## 3. The Fish Embryo Acute Toxicity Test as a Method to Determine the Lethal Effect of Environmental Pollutants

The assessment of acute toxicity provides information on the adverse effects of the test chemicals after immediate or short-term exposure. Measurement of the lethal concentration 50 (LC50) is one of the endpoints used in chemical screening for acute toxicity in the zebrafish model [33,62]. The LC50, by definition, is the amount of a test substance estimated to be lethal to 50% of the test animals within a specific duration of exposure [63]. The fish embryo toxicity test (FET) has been approved by the working group of national coordinators of the OECD as a tool to determine the LC50 in embryonic zebrafish [64]. Briefly, fertilized zebrafish eggs (before the 16-cell stage) are collected and are immediately exposed to the test solution until 96 hpf. Fertilized eggs are screened with stereomicroscopy and transferred to 24-well plates. The test medium is renewed daily, and the lethal endpoints are recorded from 24 to 96 h. The lethal endpoints specified by the OECD include coagulation of the embryo (24–96 h), lack of somite formation (24–96 h), non-detachment of the tail (24–96 h), and lack of heartbeat (48–96 h). At the end of the exposure, acute toxicity and the LC50 are determined by cumulative mortality.

The FET is a good surrogate for rodent models and the AFT [64,65,66]. For example, Ali et al. suggested zebrafish embryos are a good predictive model for evaluating toxicity due to a strong correlation between the zebrafish LC50 from the FET and the rodent lethal dose 50 (LD50) over 60 test chemicals [32]. Lammer et al. found that both the FET and the AFT provided highly similar results in an evaluation of 143 test substances [66]. The FET appears to be a valuable assay in determining LC50 from test chemicals; however, the characteristics of the test chemical and limited bioavailability capacity in embryonic zebrafish may affect the applicability of the FET [67]. For example, the chorion is fenestrated with 0.5 µM diameter pores that allow the passage of small molecules, including water, ions, and chemicals; however, it may act as a barrier for chemicals with a high molecular weight (≥3 kDa) or molecules with solvents, side chains, and electric charges [33]. Although many studies support embryonic zebrafish as a more sensitive model than adult fish, zebrafish at the early stage may have limited bioavailability or biotransformation capacity for specific chemical compounds [68]. For example, Kluver et al. indicated that some neurotoxic compounds were more toxic in adult zebrafish than embryonic zebrafish [68]. Glaberman et al. found that the embryo-based test had a lower sensitivity for neurotoxic pesticides than the juvenile fish toxicity test [69]. Additionally, it has been argued whether using chorionated or dechorionated zebrafish embryos benefits the FET [70]. Dechorionation has been suggested to improve the results of the FET but dechorionation is often associated with lower survival rates [70]. Olivares et al. indicated that the removal of the chorion in arsenic-exposed zebrafish embryos increased mortality and developmental abnormalities at 120 hpf [71]. However, Coral et al. evaluated the arsenic bimodal concentration responses in embryonic zebrafish and found both dechorionated and chorionated zebrafish embryos had similar findings at 96 hpf [72].

## 4. Investigating the Exposure to Environmental Pollutants as a Risk Factor for Neurodegenerative Disorders in the Zebrafish Model

Neurodegenerative disorders encompass a broad range of conditions characterized by progressive loss of selectively vulnerable cells in the nervous system that are essential for movement, coordination, and cognition [73]. Current studies have linked environmental pollutant exposure to neurodegenerative disorders, with the majority focusing on unraveling the underlying mechanisms [74]. Studies have shown that neurodegenerative disorders are multifactorial and involve a relatively complex and overlapping network [6]. Most studies suggest that environmentally induced oxidative stress, apoptosis, altered neurotransmission, and epigenetic modification contribute to neurodegenerative disorders [75,76]. Among these, oxidative stress is proposed to be the central regulator in response to environmental stressors and leads to neurologic deficits [77,78].

AD is the most prevalent neurodegenerative disorder that primarily affects the elderly population [79]. Clinical manifestations of AD patients include dementia, impaired learning, and progressive cognitive dysfunction [80,81]. The hallmark of the neuropathologic finding is the intraneuronal and extracellular amyloid β (Aβ) aggregates accompanied by intracellular neurofibrillary tangles (NFTs) composed of hyperphosphorylated tau protein, distributed throughout the hippocampus, temporal lobe, frontal lobes, and frontal cortex of the brain [82]. The pathogenesis of AD is still unclear; however, oxidative stress has been considered as the primary contributor in AD patients [79]. Oxidative stress induces mitochondrial dysfunction, triggers the caspase pathway, and leads to cellular apoptosis [83]. Oxidative stress also promotes the production of hyperphosphorylation Tau protein by down-regulating protein phosphatase 2A (PP2A), a major tau phosphatase in the human brain [84]. Moreover, oxidative stress impairs the proteasome, results in protein misfolding, and leads to Aβ plaque accumulation [81]. Furthermore, studies have shown that oxidative stress mediates increased levels of neuroinflammation with microglial cell activation [85,86]. Collectively, oxidative stress facilitates cellular apoptosis, the formation of Aβ plaques, hyperphosphorylation of the Tau protein, and neuroinflammation that prompts AD development [79].

Metal exposure has been proposed as having a role in AD patients [74]. Increased amounts of copper, zinc, iron, aluminum, and mercury have been detected in the serum and brain of AD patients [74]. Aluminum has the potential for neurotoxicity and strongly correlates to neurodegeneration [87]. Studies reported a higher incidence rate of AD patients in the areas where the drinking water detected more elevated levels of aluminum [88,89]. The Tau mouse model with 2 mM orally administrated aluminum progressively showed tau accumulation, apoptosis, and neurologic dysfunction [90]. It is suggested that an excess glutamate level induces neuronal cell damage by generating free reactive radicals and inhibiting antioxidant enzymatic activity. Kaur et al. investigated aluminum-induced neurotoxicity in the zebrafish model and found zebrafish with 200 mg/kg daily exposure to AlCl3 had increased oxidative stress, reduced antioxidant levels, and altered neurotransmitters, and exhibited learning and memory deficits [91]. Lee et al. suggested other metals, such as lead, trigger AD by identifying sortilin-related receptor, L (DLR class) A repeats-containing (SORL1), an AD genetic risk factor, in the lead-exposed embryonic zebrafish [92].

PD is the second most common neurodegenerative disorder that begins in mid to late life [93]. PD patients show typical symptoms such as bradykinesia, slow movement, and difficulties in fine motor control [94]. Degeneration and loss of dopaminergic neurons in the substantia nigra and the deposition of Lewy bodies containing α-synuclein in the less-affected dopaminergic neurons are the main pathologic findings reported in PD patients [95]. Diagnostic biomarkers for PD have been proposed, such as DNA oxidative products, reduced proteasome 20s activity, increased caspase-3, 8, and 9 expression, reduced dopamine levels, decreased expression of dopamine type 3 receptor (D3R), and the presence of α-synuclein [8,96,97,98,99].

While the underlying mechanism remains obscure, it is proposed that, similar to that of AD, oxidative stress, dysfunctional mitochondria, apoptosis, neuroinflammation, altered neurotransmission, and antioxidant deficiency in response to environmental pollutants play a role in the dopaminergic neuronal cell death in the substantia nigra of PD patients [77,100]. For example, rotenone is a historically used pesticide [100]. Rats exposed to rotenone develop an impaired mitochondria complex I, in which the dysfunctional mitochondria then reduces ATP production, triggering the formation of reactive oxygen species, and resulting in cellular apoptosis and a dysfunctional proteasome, leading to cytoplasmic accumulation of α-synuclein [101]. These findings largely resemble that of the mechanism found in human PD patients. Similar findings were also observed in Fischer 344 rats orally administrated 500 and 1000 mg/kg Trichloroethylene (TCE), an industrial degreasing agent, for six weeks [102]. Rats had significantly reduced mitochondrial activity with increased oxidative stress and intracellular α-synuclein accumulation. Wang et al. found 10 and 100 µM paraquat, a dipyridyl herbicide, impaired mitochondrial function, up-regulated oxidative stress-related genes, altered dopamine signaling genes, and increased locomotor activity in the zebrafish larvae at 7 dpf after exposure for 96 h [103]. Additionally, propamocarb, a systemic fungicide, is linked to neurodegenerative disorders in the larval zebrafish through abnormal swimming behavior in response to light–dark changes after exposure at the concentration of 100 and 1000 µg/L for 96 h [104]. In this study, the authors also found that propamocarb exposure affected antioxidant and dopaminergic enzymatic activity and neurotoxicity-related genes. Besides pesticides, Kayln et al. reported that embryonic zebrafish exposed to anthropogenic fluorosurfactant, perfluorooctanesulfonic acid (PFOS) (0.1 mg/L), 6:2 chlorinated polyfluorinated ether sulfonate (F-53B) (1 mg/L), and sodium p-perfluorous nonenoxybenzene sulfate (OBS) (1 mg/L), from 72 to 120 hpf, showed hypo-locomotion, reduced subpallial dopaminergic neurons, and reduced dopamine transporter expression [105].

Diagnosing neurodegenerative disorders remains challenging. Patients with neurodegenerative disorders at an early stage frequently show subtle and unclear symptoms that may overlap with other neurodegenerative conditions [106]. Validating diagnostic biomarkers is, therefore, essential for early diagnosis. Several biomarkers related to oxidative stress, such as elevated levels of oxidative products, increased reactive oxygen species, and altered levels of antioxidants and neurotransmitters, have been widely used for detecting AD and PD.

### 4.1. Oxidative Stress

Oxidative stress reflects an imbalance between the production of reactive oxygen species (ROS) and antioxidant defenses in the body [83]. Excessive ROS can potentially cause severe damage to cells [83]. ROS can be produced endogenously in cells as a natural by-product of normal cellular metabolism, including superoxide anion (O^−2^), hydrogen peroxide (H_2_O_2_), and hydroxyl radical (HO•) [107,108]. Recent studies indicated that environmental pollution such as cigarette smoke, ozone exposure, ionizing radiation, stress, and heavy metal ions generate exogenous free radicals [109]. Vertebrates are equipped with antioxidant systems, such as superoxide dismutases (SOD), catalase (CAT), glutathione peroxidase (GPx), glutathione reductase (GR), and glutathione (GSH) to counteract the adverse oxidant effects [83]. However, when oxidative status is disrupted, ROS attack nucleic acid, lipids, and proteins, resulting in DNA base oxidation, lipid peroxidation, and protein carbonylation, respectively. The brain is highly vulnerable to oxidative stress, having a high rate of oxygen utilization and relatively low concentrations of antioxidants and associated enzymes, and, in addition, it contains a high content of polyunsaturated lipids and the biomacromolecules that are most susceptible to oxidation [78,110]. Moreover, substantial nigral neurons appear more vulnerable to oxidative stress than other brain regions due to their much lower antioxidant activity and higher metabolic rate [111,112].

#### Zebrafish as a Model in Oxidative Stress Studies

Zebrafish at different life stages, from embryos to juvenile to adult, have been used to evaluate oxidative stress in vivo [113]. Zebrafish contain similar ROS-based signaling and defense mechanisms against environmental chemicals, including oxidants and electrophiles, to those of mammals. Many studies have adopted the zebrafish model to investigate environmental pollutant-induced oxidative stress.

The evaluation of oxidative stress is important for studying environmentally induced neurotoxicity. Many approaches have been designed to directly or indirectly measure oxidative damage [108,114]. An increase in ROS reflects an uncontrolled oxidative status; however, it has long been a challenge to directly analyze ROS in cells because ROS are short-lived (milliseconds or less), rapidly altered, and have low steady-state levels (picomolar to low micromolar) [108]. Conventional studies utilize dichlorodihydrofluorescein diacetate (H_2_DCFDA) as a chemically reduced indicator to detect cellular production of ROS [115]. In brief, this assay requires a membrane-permeable fluorogenic probe H_2_DCFDA to diffuse into targeted cells and transform to an oxidant-sensitive compound, 2′,7′-dichlorodihydrofluorescein (H_2_DCF), through deacetylation in reaction to cellular esterase [116]. Cellular ROS rapidly oxidize H_2_DCF to a highly fluorescent compound, 2′,7′-dichlorofluorescein (DCF), for detection. H_2_DCFDA has been reported to detect hydrogen peroxide, hydroxyl radicals, and peroxyl radicals by measuring the fluorescence intensity as analyzed by flow cytometry or by a fluorescence plate reader [117,118]. Yang et al. found that after exposure to isoprocarb, a carbamate insecticide, at the concentrations of 1, 1.75, and 2.5 mg/L, the embryonic zebrafish had elevated ROS levels by increasing H_2_DCFDA fluorescence signals after 72 h exposure [119].

Researchers are also inclined to search for more stable and enduring biomarkers for oxidative damage assessment. Although these alternative methods may not fully represent the cellular ROS status, they are promising as ways to evaluate oxidative damage by indirectly measuring oxidative products from DNA/RNA, lipids, and protein in cells. These biomarkers include DNA/RNA oxidation, lipid peroxidation, and protein carbonylation [108].

Oxidative nucleic acid modifications are frequently used as markers to evaluate oxidative damage. The comet assay and 8-oxo-7, 8-dihydro-2′-deoxyguanosine (8-oxo-dG)/8-hydroxy-2′-deoxyguanosine (8-OHdG) are two widely studied methods [120,121]. The comet assay, also called single-cell gel electrophoresis, is a method to measure the general cellular oxidative damage to DNA by detecting DNA strand breaks and alkali-labile sites [122]. Some research has focused on measuring the level of 8-oxo-dG or 8-OHdG, pivotal oxidative products of DNA, with several techniques such as ultraperformance liquid chromatography–mass spectrometry (UPLC-MS), enzyme-linked immunosorbent assay (ELISA), and high-performance liquid chromatography (HPLC) coupled with an electrochemical detector. Lipid peroxidation has been commonly used to indicate cell membrane damage in response to ROS-mediated injury [123,124]. Malondialdehyde (MDA) is one of the lipid peroxidation end-products that is well-studied, relatively stable, and often considered a good index to estimate oxidative stress levels by reacting with TBARS (thiobarbituric acid reactive substances) [123,125]. The TBARS assay is widely applied to measure lipid peroxidation in cells, tissue, and biological fluids. It is designed to detect the reaction of lipid peroxidation products, primarily malondialdehyde (MDA), with thiobarbituric acid (TBA) [125,126]. This end product, MDA-TBA2 adducts, also called TBARS, can be detected spectrophotometrically by colorimetric or fluorometric plate readers. Measuring the protein carbonyl level is another common approach for oxidative damage evaluation. Carbonyl groups are produced during protein carbonylation by oxidizing the protein backbones and amino acid residues such as proline, arginine, lysine, and threonine. Carbonylated proteins are reported to have relative early formation and stability compared with other oxidative products and are thus frequently used in oxidative stress studies. Detection of protein carbonylation involves the reaction with 2,4-dinitrophenylhydrazine (DNPH) with the formation of stable dinitrophenylhydrazone products, which then can be analyzed by a variety of means such as spectrophotometric assay, ELISA, and one-dimensional or two-dimensional electrophoresis followed by a Western blot immunoassay. Zhu et al. studied the effects of developmental exposure to the SiO_2_ in the zebrafish model [127]. They found that zebrafish larvae exposed to 100 mg/L SiO_2_ for 5 days had significant changes in ROS levels, increased 8-OHdG content, and decreased GSH. An amount of 100 µ/L bisphenol A has been found to trigger oxidative stress in 120 hpf zebrafish by observing an increase in fluorescence lipid peroxidation after 24 h exposure [128]. Butylated hydroxyanisole (BHA), a widely used chemical in the cosmetics, pharmaceutical, and food industries, has been reported to trigger ROS-induced apoptosis in zebrafish larvae after 96 h exposure to positive 8-OHdG immunofluorescence of the treated group with 1, 5, 7.5, and 10 ppm [129].

Balanced redox homeostasis requires a large amount of antioxidants to counteract ROS from causing cell damage. A variety of direct or indirect methods of measuring antioxidants are available. For example, many commercial superoxide dismutase activity kits are produced according to Beauchamp and Fridovich’s method. Briefly, this method measures SOD enzymatic activity by indirectly monitoring the reduction in nitroblue tetrazolium (NBT) [130]. Superoxide ions are generated from the xanthine–xanthine oxidase system and can convert NBT to NBT-formazan. The SOD in the test sample, therefore, can compete with the superoxide ions and lowers the rate of NBT-diformazan conversion. The decreased signal determines the SOD inhibition activity. CAT can be quantified by observing the decrease in hydrogen peroxide through the spectrophotometric method [131,132]. Glutathione peroxidase converts glutathione (GSH) to glutathione disulfide (GSSH) [131]. The GSH/GSSG ratio is commonly accepted as an indicator that reflects cellular redox homeostasis. Sun et al. exposed zebrafish to 0, 25, 50, 75, or 150 μg/L AsIII until 120 hpf and their results indicated that a low concentration of AsIII induced oxidative stress by detecting elevated SOD, altered Cu/ZnSOD and MnSOD mRNA transcriptional levels, and increased malondialdehyde levels [133]. Adeyemi et al. found increased levels of lipid peroxidation and DNA oxidation. The decreased GSH and catalase activity was observed in 96 hpf zebrafish embryonically co-exposed to atrazine (0.1 mM) and arsenic (0.8 mM) by utilizing TB, and spectrophotometric methods [134].

Several transgenic zebrafish models have also been employed in the study of oxidative stress [135,136]. Nuclear factor erythroid 2-related factor (Nrf2) is a key transcription factor regulating the cellular response against oxidative and nitrosative stress and has been used as a marker for monitoring oxidative stress [137]. Liu et al. successfully created the nrf2a-eGPF zebrafish model to evaluate PCB126-induced oxidative stress-mediated apoptosis and developmental toxicity [135]. Kusik et al. used the EPRE-LUC-GFP zebrafish model to study mercury-induced oxidative stress [136]. Finally, Mourabit et al. developed the first stable transgenic zebrafish line Tg(3EpRE:hsp70:mCerry) to investigate the imbalance cellular redox against various environmental conditions [138].

### 4.2. Apoptosis

Apoptosis, also known as programed cell death, is a pathway of cell death associated with normal eukaryotic development and the maintenance of organismal homeostasis [139,140]. The pathway is controlled by several families of proteins and is classified into two pathways: intrinsic (mitochondrial) and extrinsic (death receptor) [141]. Viable cells are sustained by receiving survival signals, such as growth factors, to induce the production of anti-apoptotic proteins (BCL2, BCL-XL, and MCL1) that prevent cytochrome c leakage from mitochondria. In the intrinsic apoptotic pathway, conditions such as growth factor withdrawal, DNA damage, and protein misfolding induced by endoplasmic reticulum stress activate cellular sensors to antagonize anti-apoptotic proteins and activate pro-apoptotic proteins, BAX and BAK, to form channels on the outer mitochondrial membrane and release cytochrome c, triggering caspase cascades. The extrinsic pathway employs death receptors that deliver an apoptotic signal, leading to autocatalytic caspase activation through initiator caspase 8, 9, and 10 and executioner caspases 3 and 6 [142]. Following the apoptotic pathway, the cells become shrunken with chromatin condensation, membrane blebbing, and nuclear fragmentation [141].

#### Zebrafish as a Model in Apoptosis Studies

The zebrafish is a useful experimental animal model to investigate apoptosis in vivo [143]. A variety of methods have been designed to evaluate apoptosis in zebrafish studies [144,145]. Several staining methods are used to detect apoptosis in tissue sections [146]. Acridine orange (AO), Hoechst staining, Annexin V, DNA ladder, Terminal deoxynucleotidyl transferase dUTP nick end labeling (TUNEL) assay, Caspase-3/7 activity, and ssDNA staining have been described [147,148,149,150,151,152].

AO stain, first described by Strugge and Hilbrich in 1941, is a nucleic acid selective metachromatic fluorescent dye that permeates both live and dead cells and preferentially binds to nucleic acid, but particularly stains apoptotic nuclei [153,154]. AO stain is one of the most commonly used techniques, which can be performed rapidly on embryonic zebrafish in multiwell plates [155]. Parlak et al. observed zebrafish larvae at 96 hpf that were oxidatively stressed with decreased SOD, CAT, GPx, increased MDA and ROS, and elevated apoptosis signaling, highlighted with AO stains after 96 h exposure to 50 mg/L deltamethrin [156]. Embryonic zebrafish exposed to 400 nM methylmercury had increased AO-positive apoptotic cells detected in the brain at 96 hpf [157]. Similarly, 9 μM cadmium-induced apoptosis was detected by AO stain at 24 hpf [158].

The TUNEL assay has been considered a sensitive, quantitative, and universally applicable method for identifying apoptotic DNA fragmentation [148,159]. Nuclear fragmentation accompanied by 3′ hydroxyl terminus of DNA breaks is a hallmark finding of apoptosis. The TUNEL assay utilizes a unique enzyme, Terminal deoxynucleotidyl Transferase (TdT), to detect and catalyze modified fluorescent conjugated deoxynucleotides (dUTPs) that preferentially bind to DNA breaks. The catalyzed dUTPs emit a fluorescence signal and are detected by fluorescence microscopy or flow cytometry.

Studies have used monoclonal antibodies to directly detect caspase-3 in fixed zebrafish embryos [144]. With the advancement of molecular techniques, more studies have chosen quantitative polymerase chain reaction (qPCR) and whole-mount immunofluorescence to detect apoptotic-related genes [144,160]. Moreover, with the advantage of clustered, regularly interspaced, short palindromic repeats (CRISPR-Cas9) genome editing methods, multiple transgenic zebrafish lines have been created [161,162]. For example, Annexin V (A5) is a phospholipid-binding protein with a high affinity for phosphatidylserine and has been used as a maker to detect apoptotic cells [163,164]. van Ham et al. developed a secA5-YFP transgenic line by fusing secreted A5 to yellow fluorescent protein to label apoptotic cells in zebrafish embryos [161]. Synthetic zebrafish pro-caspase-3 has been microinjected into one-cell stage zebrafish embryos, and pro-caspase-3 transgenic zebrafish have been shown to be markedly sensitive to UV irradiation, which induced extensive apoptosis [162].

### 4.3. Neurotransmission

Neurotransmitters are chemical messengers carrying information between neurons and are critical for neurodevelopment, learning and memory, and behavior [165,166,167]. The neurotransmitter system is conserved throughout vertebrates [168]. Major neurotransmitters such as glutamate, GABA, catecholamines, serotonin (hydroxytryptamine or 5-HT), and histamine systems have been well-described [166]. Exposure to environmental stimuli, drugs, chemotherapeutic agents, radiation, or food additives is linked to neurodegenerative diseases and neurologic disorders by affecting the synthesis, storage, and release of neurotransmitters [46,169,170].

Glutamate is the primary excitatory neurotransmitter that regulates synaptic transmission and neuronal excitability [171,172]. Glutamate is important for memory, cognition, and mood regulation and is associated with neurodegenerative diseases [171,173]. Conversely, GABA is the major inhibitory neurotransmitter and is widely distributed throughout the brain [174,175]. GABA modulates post-synaptic receptor activity, hyperpolarizes the cells, and inhibits the transmission of action potentials [176,177]. Impaired GABA signaling is linked to multitudes of neurologic and psychiatric disorders [178,179,180]. Catecholamines are monoamine neurotransmitters that consist of a group of chemicals with hydroxyl groups on a benzene ring, in which dopamine, norepinephrine, and epinephrine are the main neurotransmitters, which are associated with movement, memory, and depression [181]. The histaminergic system exerts effects on memory, cognition, circadian rhythm, and feeding and drinking [182,183,184,185]. Finally, serotonin (5-HT) is a biological amine and is implicated in neurodevelopment, neuroendocrine function, mood, and circadian rhythms [186,187,188].

#### Zebrafish as a Model for Neurotransmission

The neurotransmitter system is highly conserved between zebrafish and mammals [46]. Zebrafish share the common neurotransmitter pathways with mammals, making the zebrafish model a powerful tool for studying mechanisms of chemical neurotoxicity [60,189]. Neurotransmitters are mainly measured and quantified by HPLC coupled with fluorescence, optical density, mass spectroscopy, luminescence, or electrochemical methods [190]. Tufi et al. detected and quantified multiple neurotransmitters by hydrophilic interaction liquid chromatography (HILIC) coupled to tandem mass spectrometry (MS/MS) and further used this analytical method to study neurotransmitters in zebrafish embryos [191]. Several molecular techniques have been used to evaluate gene expression associated with neurotransmitters, such as qPCR, microarray, RNA-seq, and next-generation technologies [46,192,193].

Tufi et al. first used HILIC to evaluate neurotransmitters in the zebrafish model [191]. Eight neurotoxic pesticides were selected and exposed to embryonic zebrafish for 5 days, and a significant change in neurotransmitter levels during early zebrafish development was observed in their study. Wirbisky et al. investigated GABA during the early life stage of zebrafish with lead exposure [194]. HPLC results showed increased GABA levels at 48 hpf in the 10 and 50 ppb treatments but revealed a significantly decreased GABA level at 72 hpf in all treated groups (10, 50, and 100 ppb). Altered GABAergic pathway gene expression was also detected during the study. Kanungo et al. found that zebrafish embryos exposed to inorganic arsenic had reduced acetylcholinesterase (AChE) activity, altered tyrosine hydroxylase positive (TH) (dopaminergic) neuron development, and increased spinal motor neurons in 72 hpf zebrafish larvae with 200 and 400 mg/L exposures [195]. Finally, Ding et al. found that 10–100 μg/L photoaged microplastics, ubiquitous neurotoxic environmental contaminants, induced neurotoxicity through oxidative stress and abnormal neurotransmission in the zebrafish at 120 hpf by observing significantly altered antioxidant enzymes, oxidative products, and increased levels of neurotransmitters [196].

### 4.4. Epigenetic Modification

Epigenetics involves the molecular modification of genes and proteins that occurs without changing DNA’s primary structure. DNA methylation, histone modification, and non-coding RNA (ncRNA) are the three most studied epigenetic mechanisms [197].

DNA methylation is a heritable epigenetic modification and has been most intensely investigated [198]. DNA methylation is essential for gene expression, genomic imprinting, embryonic development, and memory formation and storage [199]. The principal role in regulating gene expression of DNA methylation is adding methyl groups to the C5 position of the cytosine ring of DNA, forming 5-methylcytosine (5mC) [35]. In mammals, this process frequently occurs within CpG islands through a group of DNA methyltransferases (Dnmts). Several Dnmts have been discovered, with Dnmt1, Dnmt3A, and Dnmt3B most frequently discussed [200]. Dnmt1 is a major enzyme mainly responsible for DNA methylation and is involved in inheritance. Dnmt3A and Dnmt3B are predominantly associated with de novo methylation. Hypermethylated genes with the presence of mC typically result in transcriptional silencing by forming compacted and closed chromatin. However, ten-eleven translocation (TET) enzymes can actively demethylate genes by oxidizing 5mC to 5-hydroxymethylcytosine (5hmC), 5-formylcytosine (5fC), and further to 5-carboxycytosine (5caC) and activate gene transcription.

Histone modification is one of the most important reversible epigenetic modifications. Histone modification has been discovered as a regulator in neurogenesis and neurodegenerative and neuropsychiatric disease development [201,202,203]. Mechanisms of histone modification include methylation, acetylation, biotinylation, SUMOylation, phosphorylation, and other chemical modifications that alter the structure of the chromatin or histone core. Enzymes such as histone-deacetylases (HDACs), histone-acetyltransferases (HATs), and histone-methyltransferases (HMTs) are essential in transcriptional regulation [202]. Four core histone proteins (H2A, H2B, H3, and H4) are wrapped by 147 bp of DNA and stabilized by a linker histone (H1). N terminal histone tails and histone globular domains are locations frequently targeted. Histone methylation with methylated lysines or arginines may be associated with either transcriptional activation or repression. Histone acetylation with lysine residues acetylated by HAT leads to increased transcription. On the other hand, chromatin condensation can occur with histone deacetylation by HDAC.

Non-coding RNAs (ncRNAs) have been implicated as critical epigenetic regulators in posttranscriptional silencing [204]. Regulatory ncRNAs include micro-RNA (miRNA), small interfering RNA (siRNA), piwi-interacting RNA (piRNA), and long non-coding RNA (lncRNA) [205]. Among these, miRNA are well-described, play a fundamental role in gene regulation, and are linked to normal developmental pathways and pathologic conditions. miRNAs are single-stranded, relatively short, and approximate 18–22 nucleotides. miRNAs are formed from long double-stranded RNA enzymatically processed into smaller fragments by RNase III enzymes (DICER). Repression on targeted genes occurs when the miRNA and RNA-induced silencing complex (RISC) detects an improper base pairing.

#### Zebrafish as a Model of Epigenetic Modification

Many studies indicate that zebrafish are good models for studying epigenetics [206]. First, with genome editing becoming more mature and prevalent, such as zinc finger activator-like effector nucleases (TALENs) and CRISPR/Cas9, the design and generation of mutants of proteins involved in the epigenetic process have become possible [207,208,209]. Second, zebrafish contain eight DNA methyltransferase “orthologous to” mammalian DNMTs [210]. Third, zebrafish do not have genome imprinting; rather, different from mammals and other species of fish, zebrafish have maternal genome reprogramming with passive demethylation during early development, whereas the paternal zebrafish genome is resistant to demethylation [210,211]. Therefore, many genes involved in the epigenetics process in the early embryos are maternally transferred, and prolonged survival is possible in these mutants during the first stage of development.

Numerous methods of evaluating epigenetic modifications have been designed [212]. For example, conventional techniques such as bisulfite genomic sequencing analysis are used for analyzing DNA methylation [213,214]. In brief, this method utilizes sodium bisulfite to convert cytosine to uracil in single-stranded DNA and recognized as thymine in subsequent PCR amplification and sequencing; however, 5mCs are spared this conversion and remain as cytosine, allowing 5mCs to be detected from unmethylated cytosine [213,214,215]. Other emerging tools, such as global analysis and third-generation sequencing-based technologies, are also frequently applied [216,217,218]. Chromatin immunoprecipitation (CHIP) assay is a commonly used method for detecting histone modification [219]. This specific antibody-directed ChIP assay is a particularly useful technique in studying DNA–protein interactions that allows the chromatin structure surrounding a particular DNA sequence to be analyzed [220,221]. Novel methodologies have been developed to identify chromatin occupancy sites using immunoprecipitation-based approaches followed by next-generation sequencing (NGS) [222].

It is widely accepted that stimuli such as changes in environment, nutrition, and chemical compound exposure trigger epigenetic modifications [223,224]. For example, Li et al. used a 5-methylcytidine-specific antibody to assess global methylation on zebrafish that were exposed to sodium arsenite at the embryonic stage [129]. They found zebrafish larvae at 24 and 48 hpf had abnormal genomic DNA methylation patterns with decreased methylation in the brain with exposure to 2 mM sodium arsenite. Bian et al. investigated DNA methylation and gene expression in cadmium-exposed embryonic zebrafish [225]. They targeted global methylation and DNMT levels in 24 hpf zebrafish and observed a significantly up-regulated *dnmt1* expression and down-regulated *dnmt3* and global methylation at 0.89 μM. Wirbisky et al. utilized a microarray to analyze zebrafish larvae at 72 hpf and found zebrafish in the 30 ppb-treated group had altered miRNA related to neurodevelopment, differentiation, and maturation after atrazine exposure [226]. Wang et al. used whole genome bisulfite sequencing to assess the neurologic effects of atrazine exposure on DNA methylation in female zebrafish that were exposed at embryonic stages [223]. Their results demonstrated that atrazine at 30 ppb had the most increased methylation of genes associated with neurological pathways.

## 5. Zebrafish Neurobehavior

Neurobehavior evaluation is one of the most sensitive methods of neurotoxicity assessment [227]. Zebrafish behavior is becoming increasingly popular as a measurable phenotype reflecting alterations in normal physiology [228]. The deviated behavior may represent possible functional outcomes of chemical toxicity, disruption of neurotransmission, altered intracellular signaling, abnormal musculoskeletal system, and altered growth and development [36,191,229]. In addition, zebrafish models have frequently been used to evaluate neurotoxic compound-associated neurobehavioral changes, such as locomotor activity, anxiety, agitation, sedative effects, and learning and memory behaviors [230].

### 5.1. Zebrafish Embryonic and Larval Locomotor Activity

Zebrafish embryos begin spontaneous muscle contractions at 18 hpf [231]. Embryos start to twitch in response to mechanical stimulation at 24 hpf [232]. By 2 dpf, embryos present tail-flip and fast escape response to touch stimuli [233,234]. Zebrafish develop mature swimming at 4–5 dpf, following the inflation of the swim bladder [235]. Zebrafish at early life stages exhibit a broad range of behavioral phenotypes, such as the optomotor response, optokinetic response, photomotor response (PMR), spontaneous activity, prey capture, startle response, thigmotaxis, habituation, and visual motor response (VMR) [37,126,236,237,238]. Among these, the PMR and VWR are frequently used neurobehavioral endpoints in the zebrafish model [239,240,241].

The PMR is a nonvisual, robust, reproducible behavioral response to intense photic stimuli by activating light-sensitive neurons in the hindbrain of the developing zebrafish embryos [242]. Briefly, at 24–30 hpf, zebrafish embryos during the PMR test are kept in the dark with two light pulses. Following the light stimulus, zebrafish embryos go through three phases: the latency phase, excitation phase (PMR phase), and refractory phase [237,243]. The changes in movement in response to light stimuli have been reported to be related to increased relative risk for developmental malformations at 120 hpf [240,241].

The VMR, compared to the PMR, is a visual motor response provoked by changes in ambient illumination [244]. Zebrafish larvae make frequent and low-amplitude propulsive and turning movements at stable lighting conditions but present a high-angle turn, followed by a propulsive movement at an abrupt transition from light to dark [239,244]. The altered locomotor activity may reflect impaired brain function, neurodevelopment, neurotransmission, or visual pathways [244].

Many studies have utilized the PMR and VMR in investigating environment-induced neurotoxicity [245,246]. For example, Horzmann et al. found that zebrafish embryos exposed to TCE showed altered behavioral responses during PMR and VMR assays after exposure to 5, 50, and 500 ppb for PMR, and 500 ppb for VMR assays for 72 h [245]. Olivares et al. detected hypoactive locomotor PMR in the zebrafish larvae exposed to 900–1000 mM arsenic [71]. Perfluoroheptanoic acid (PFHpA) is an organic molecule with fluorinated carbons used in many consumer products [247]. Huang et al. found that zebrafish embryos exposed to 0.1 µM PFHpA for 7 days showed developmental and mitochondrial toxicity and reduced locomotor activity on the VMR [246]. Gyimah et al. found that Bisphenol AF (BPAF), a chemical widely used in manufacturing, induced oxidative stress and apoptosis in the zebrafish model [248]. They further assessed locomotor activity on larvae at 120 hpf by VMR in 0.1, 0.3, and 1.0 μM treatment groups, and their findings indicated that BPAF induced a dramatic reduction in locomotor activity. Lockwood et al. demonstrated a hypoactive locomotor response with sedative effects at 4% ethanol after exposure to ethanol in larval zebrafish [249].

Zebrafish larvae are sensitive to neuroactive agents, and it is suggested that they have locomotor activity responses similar to those of mammals [250]. Some neurotoxic substances were found to be less acutely toxic to the zebrafish embryos than to the adult fish; however, Kluver et al. found zebrafish larvae exhibited altered locomotor activity in response to the concentrations of neuroactive compounds that caused lethality in adult zebrafish [68]. Therefore, their studies suggested the alteration of locomotor in zebrafish larvae could improve the prediction of acute toxicity for neurotoxic compounds in adult zebrafish.

Although in our review article we mainly focus on investigating the early-life zebrafish exposed to environmental pollutants, several studies emphasize the effect of early-life exposure-induced long-term neurotoxicity in adult zebrafish.

### 5.2. Adult Zebrafish Behavior

Adult zebrafish have a higher complexity of behavioral responses than those in the early stage of zebrafish [228,251]. For example, associative learning is described in adult zebrafish but has yet to be established in zebrafish larvae [252,253]. Adult zebrafish behavioral assays have frequently been used for evaluating locomotor activity, sedation, agitation, anxiety, learning and memory, social reaction, and changes after xenobiotic exposure [251]. Neurobehavioral tests usually require video tracking software to analyze parameters such as velocity, movement, mobility, total distance moved, and latency to first zone entry [254,255].

Several behavioral assessment tests in the adult zebrafish model have been adapted from rodent models and are used in neurotoxicity studies, such as the novel tank test, open field test, inhibitory avoidance test, novel object recognition test, maze test, shoaling test, and light and dark test [36]. For example, the novel tank and open field tests in the zebrafish model are neurobehavioral tests similar to rodent open field tests for assessing anxiety-related behavior [256,257]. Researchers use maze tests to evaluate rodent cognition deficits in the zebrafish model as well [258,259].

Few studies have investigated the long-term neurotoxicity in adult zebrafish exposed to neurotoxic compounds during the early life stage. In one example, Wang et al. investigated the continuance of the toxic effects of lead on zebrafish neurobehavior from larval to adult age [260]. They found that the gene expression linked to neurodevelopment and neurotransmitter systems altered in a similar pattern in both larvae and adult zebrafish. In addition, zebrafish behaviors were evaluated through the VMR, open field test, novel tank test, social interaction test, and T maze test. The result revealed consistency in locomotion parameters in both larvae and adult zebrafish, including increased distance traveling, cumulative mobility, and velocity. They, therefore, speculated that the disruption of early neurodevelopment, as well as prolonged modulation of neurotransmitter systems, contributed to the lead-induced neurobehavioral disorders observed in juveniles and adults. Moreover, embryonic exposure to methylmercury had been found to alter locomotor activity and anxiety-related responses, in addition to dopamine level changes in both larval and adult zebrafish [261]. Although several zebrafish neurobehavior assays have been proven to provide a phenotypic outcome of neurotoxicity and are useful to assess xenobiotic exposure, the limitation of a lack of standardization in methods has long been a concern.

## 6. Limitations in Using Embryonic Zebrafish as Models in Assessing Neurotoxicity Associated with Environmental Pollutants

Although the embryonic zebrafish as a model provides a powerful platform for neurotoxicity biomedical research, interspecies differences should be considered when translating the findings in zebrafish to humans. For example, despite genetic similarities to humans, zebrafish genes are duplicated, which increases the genetic variability and the challenge of investigating genomic function [41]. Such variability and species differences may influence the neurotoxic effects. The nervous system in early-stage zebrafish may not fully represent the neuroanatomy and neural functions of adult zebrafish or humans [262]. For example, embryonic zebrafish have an incomplete blood–brain barrier, which may result in neurotoxic compound penetration differences [55]. In addition, while the transparency of zebrafish at the early developmental stages enhances neuroanatomic studies and gains valuable insights into fields of neurodevelopment, neurologic functions, and neurodegenerative diseases, the embryonic zebrafish brain is less complex and lacks specific neural structures compared to humans, and therefore, it is crucial to consider the variation when interpreting the zebrafish findings and translating to humans [262]. Neurobehavioral assessments in embryonic zebrafish are limited by a small range of measurable behaviors compared to adult zebrafish; in addition, more complex neurobehavior, such as cognitive function, may be less sophisticated compared to adult stage or mammalian models [36]. Zebrafish possess metabolism and pharmacokinetics similar to humans in drug absorption, distribution, metabolism, and excretion; however, while using zebrafish as the model for evaluating neurotoxic compounds, the difference in metabolite processing and enzymatic activity between zebrafish embryos, later developmental stages, and humans will need to be taken into consideration during interpretation. For example, the metabolic pathway of methylmercury in humans mainly involves cysteine and glutathione, particularly in the process of detoxification [263], whereas glutathione S transferase and metallothioneins have been considered the important detoxify enzymes in the metabolic processing pathway of methylmercury in zebrafish, which may lead to variations in toxic efficacy between species [264].

## 7. Future Direction

Public awareness of environmental concerns including widespread chemical contamination has increased over the past few years and studying the potential neurotoxicity associated with environmentally relevant concentrations of pollutants is critical to understanding potential human health effects. While most studies emphasize evaluating the effects of early life exposure to neurotoxic compounds in the zebrafish model, fewer studies have investigated the neurotoxicity from environmentally relevant concentrations of neurotoxic compounds (Table 1). For example, Sun et al. explored an environmentally relevant concentration of organic benzophenone-3 (BP3) and inorganic UV filters containing titanium dioxide nanoparticles’ co-exposure-induced neurodevelopment toxicity in zebrafish [265]. The US EPA-regulated maximum contaminant level (MCL) of atrazine in drinking water is 3 ppb; however, Tai et al. observed that zebrafish had hyperactive locomotion on VMR when exposed to 0.3 ppb [35]. TCE is regulated in drinking water by the US EPA with an MCL of 5 ppb. Horzmann et al. exposed embryonic zebrafish to 0, 5, 50, and 500 ppb and observed a decreased percent hatch, shorter and narrow head, and increased duration of inactivity of PMR beginning at 5 ppb [245].

## 8. Conclusions

The zebrafish model is widely accepted in neurotoxicity assessment studies. Zebrafish embryos are suitable for high-throughput systems for chemical screening under current animal welfare legislations and appear more sensitive in response to environmental chemicals than in adult zebrafish. Advances in FET tests enable fast preliminary acute toxicity results of test chemicals. Currently, existing and newly introduced chemical wastes are environmental concerns. Environmental pollutants have been strongly linked to neurodegenerative disorders in humans. Although the underlying mechanisms are relatively complex, the zebrafish model provides a platform for evaluating neurotoxicity induced by environmentally relevant concentrations of toxicants. Mechanisms that can be evaluated include oxidative stress, apoptosis, neurotransmitter systems, neurobehavior, and epigenetic modifications. Although more research is needed to better expand the zebrafish platform, the embryonic model has shown great potential for environmentally induced neurotoxicity research.

## Figures and Tables

**Table 1:** Experimental examples of developmental zebrafish exposed to environmental pollutants induced acute neurotoxicity, altered locomotor activity, neurotransmission, apoptosis, neural-related genes. Abbreviations: ↑: increase; ↓: decrease; CAT: catalase; GABA: γ-aminobutyric; GSH: glutathione; MDA: malonaldehyde; SOD: superoxide dismutase.

Environmental Pollutants		Concentrations	Exposure Duration	Results	Reference
Insecticide	Fenpropathrin	0.016 mg/L, 0.032 mg/L, 0.064 mg/L	96 hpf	Motor ability↓ Oxidative stress↑, reactive oxygen species generation↑, CAT, SOD↓ Apoptosis-associated genes in brain and heart↑ Nrf2 signaling pathway↓	[266]
	Isofenphos-methyl	2, 4, and 8 mg/L	6–96 hpf	Survival rate, hatchability, heart rate, and body length↓ Developmental malformations (uninflated swim bladder)↑ Locomotive behavior↓ Acetylcholinesterase↓ Reactive oxygen species↑, MDA↑, SOD↑, CAT↑ GSH↑ Altered apoptosis-related genes (*bcl2*, *p53*, *bax*, and *puma*)	[267]
	Bifenthrin	103.9 and 362.1 μg/L	<3–120 hpf	Tail coiling frequency↑, altered locomotor activity (distance moved↑, turn angel↓) Acetylcholinesterase and dopamine levels↓ Neurogenesis defects (shortened brain and axon widths, and demyelination of oligodendrocytes and Schwann cells) Neurodevelopment-related genes (*gap43*, *manf*, *gfap*, *nestin*, *sox2*)↑ Neurotransmitter-related genes (*nlgn1*, *drd1*, *slc6a4a*, *ache*)↓	[268]
Fungicide	Difenoconazole	0.25, 0.5, and 1 mg/L	120 hpf	Malformation rate, spontaneous movement↑ Locomotor activity↓ Dopamine and acetylcholine↓, acetylcholinesterase↑ Altered neurodevelopmental-related genes	[269]
	Fluxapyroxad	0.5, 0.75, and 1 mg/L	96 hpf	Malformations↑ (shorter body length, smaller head and eyes, yolk sac edema) Motor aberrations Dopamine level↓ Altered acetylcholinesterase and acetylcholine	[270]
Pesticide	Trichlorfon	0, 0.1, 2 and 5 mg/L	144 hpf	Survival rate, hatching rate↓, heart beat↓ Malformation rate↑ (body length↓) Locomotor activity↓ Acetylcholinesterase, acetylcholine dopamine and serotonin↓ Central nervous system-related gene (*a1-tubulin*, *mbp*, *syn2a*, *shha*, and *gap-43*)↓	[271]
	Chlorphoxim	2.5, 5, 7.5, 10, and 12.5 mg/L	96 hpf	Mortality↑, hatchability rate and heart rate↓ Pericardial edema rate↑ Swimming behavior↓ Acetylcholinesterase↓ Altered neural-related genes (*syn2a*, *gfap*, *elavl3*, *neurog*, *gap43*, and *sox19b*) Oxidative stress↑, CAT and SOD↓ Apoptotic-related gene (*bax*, *bcl2*, *p53*)↑	[272]
	Fenvalerate	0, 3.5, 7 and 14 μg/L	4–96 hpf	Survival rate, heart rate↓ Malformation rate↑ (body length↓) Neurobehavioral alterations (spontaneous movement↓ swimming distance and velocity, movement time and clockwise rotation times↓) Cholinesterase activity↓ Neurodevelopment related genes (*elavl3*, *gfap*, *gap43,* and *mbp*) Oxidative stress↑ (reactive oxygen species↑, antioxidant enzyme↓) Apoptotic-related gene (*p53*, *bcl-2*, *bax* and *caspase 3*)↑	[273]
	Emamectin benzoate (EMB)	0.1, 0.25, 0.5, 1, 2, 4 and 8 μg/mL	4–144 hpf	Hatching rate↑ Malformation rate↑ Locomotor behavior↓ Oxidative damage↑, reactive oxygen species↑, altered oxidative stress-related genes (*cat*, *sod*, and *Cu/ZnSOD*) Altered GABA neural pathway-related genes (*gat1*, *gabra1*, *gad1b*, *abat* and *glsa*) Altered neurodevelopmental-related genes (*syn2a*, *gfap*, *elavl3*, *shha*, *gap43*, and *Nrd*)	[274]
Metal and mineral	Lead	0.1, 1, and 10 μM	120 hpf	Locomotor activity↓ Apoptosis↑ Ambra1a and ambra1b (activating molecule in Beclin1-regulated autophagy)↓	[275]
	Nickel	0, 10, 50, 100, 500, and 1000 μM	144 hpf	Hatching rate, heart rate, body length↓ Tail coiling frequency↓, swimming behavior (swimming distance, velocity, cumulative mobility)↓ Microglial activation↑ Neuronal and vascular development↓	[276]
	Selenium	0.125, 0.25, 0.5, and 1 µM	96 hpf	Locomotor activity↓ Cell apoptosis↑ Neuroinflammation↑ Dopaminergic neuron, motor neuron, GABAergic neuron and neurotransmitter transport marker genes↓	[277]
Disinfectant	Halobenzoquinones	0–8 μmol/L	120 hpf	Locomotor activity↓ Dopamine, GABA↓ acetylcholinesterase↓ Neuronal morphogenesis-related genes (*gfap*, *α1-tubulin*, *mbp*, and *syn-2α*)↓	[278]
	Perfluorononanoic Acid	0.01, 0.1, 1, 10, and 100 μg/L	120 hpf	Swimming behavior↓ Acetylcholine, glutamate, 5-hydroxytryptamine, GABA, dopamine, and noradrenaline↓	[279]
		0, 100, 500, and 1000 μg/L	4–120 hpf	Mortality↑ Hatching↓ Developmental malformations (body length↓, bent spine↑, pericardial, and yolk sac edema↑) Spontaneous movement frequency↓, altered touch-evoked response, and locomotor behavior Microglial activation↑ Acetylcholinesterase↓, dopaminergic hyperactivity	[280]
	2,5-dichloro-1,4-benuinone (2,5-DCBQ)	0.2, 0.4, and 0.6 mg/L	4–120 hpf	Developmental defects (body length↓) Heart rate↓ Abnormal motor axon structure Locomotor activity↓ Neuronal development associated genes (*gfap*, *mbp*, *syn2a*, *elavl3*, *ache*, and *α 1-tubulin*)↓ Disrupted neuroactive ligand-receptor interaction and apoptotic pathway	[281]
Nanoparticles	polystyrene nanoparticles	0.2, 1, and 5 mg/L	120 hpf	Eye and head size↓, pericardial area↑ Activity and anxiety↑ (lower dose), lethargy↑ (higher dose) Burst quality↓ tail activity↑ Acetylcholinesterase↓ Altered endocrine-related gene expression	[282]
	Microplastics	0.1 to 100 μg/L	120 hpf	Swimming speed↓ Dopamine, 5-hydroxytryptamine (5-HT), GABA, acetylcholine, and related genes↑	[283]
Flame retardant	Resorcinol bis(diphenyl phosphate)	0, 0.3, 3, 90, 300 and 900 nM	2–144 hpf	Heart rates↓ Malformation rates (body length↓) Locomotor behavior↓ Acetylcholinesterase↓ Altered neurotransmitters (GABA↑, glutamate↓, acetylcholine↑, choline and epinephrine↓)	[284]
	Decabromodiphenyl ethane	50–400 μg/L	120 hpf	Swimming speed↑ Developmental defects (pericardial edema, yolk sac edema, spine bending, and tail bending) Mitochondrial dysfunction↑ GABA, 5-hydroxyindole-acetic acid↓ Glutamicacid, norepinephrine, epinephrine, and acetylcholine↑	[285]
Drug	Mirtazapine	3.9 and 43.5 ng/L	2.5–96 hpf	Heart rate↓, hatching↑ Spontaneous contraction↓, swimming frequency and swimming speed↓ Altered epinephrine and neuregulin signaling	[286]
	Flubendazole	0.05, 0.1, 0.2, 0.4, and 0.8 mg/mL	96 hpf	Survival rate, hatching rate, and heart rate↓ Developmental abnormalities (body length, head and eye size)↓ Apoptosis↑, apoptosis-related genes (*p53*, *casp3*, and *casp8*)↑ Neural differentiation-related genes (*shha*, *nrd*, *ngn1*, and *elavl3*)↓	[287]
Others	Bisphenol AF	0.03, 0.1, 0.3, and 1.0 µM	144 hpf	Locomotor activity↓ Apoptosis↑ Reactive oxygen species↑ SOD↓ CAT↓ Altered normal dopaminergic signaling and genes	[248]
	Lanthanide phosphate (TbPO4)	10, 20, and 50 mg/L	144 hpf	Mortality↓ Spontaneous tail movement (24 hpf)↓ Dopaminergic and serotoninergic signaling↓	[288]
	Octocrylene (OC)	5, 50 and 500 μg/L	3–96 hpf	Hatching rate and heartbeat rate↓ Developmental abnormalities Oxidative damage↑, antioxidant enzyme (SOD, CAT and GST)↑ Acetylcholinesterase (AChE)↓ Apoptosis↑	[289]

## Data Availability

No new data were created or analyzed in this study. Data sharing is not applicable to this article.

## References

[1] Deuschl G., Beghi E., Fazekas F., Varga T., Christoforidi K.A., Sipido E., Bassetti C.L., Vos T., Feigin V.L. (2020). The burden of neurological diseases in europe: An analysis for the global burden of disease study 2017. Lancet Public Health.

[2] Mathur S., Gawas C., Ahmad I.Z., Wani M., Tabassum H. (2023). Neurodegenerative disorders: Assessing the impact of natural vs drug-induced treatment options. Aging Med..

[3] Tiwari S., Atluri V., Kaushik A., Yndart A., Nair M. (2019). Alzheimer’s disease: Pathogenesis, diagnostics, and therapeutics. Int. J. Nanomed..

[4] Labadorf A., Choi S.H., Myers R.H. (2017). Evidence for a pan-neurodegenerative disease response in huntington’s and parkinson’s disease expression profiles. Front. Mol. Neurosci..

[5] Xie A., Gao J., Xu L., Meng D. (2014). Shared mechanisms of neurodegeneration in alzheimer’s disease and parkinson’s disease. Biomed. Res. Int..

[6] MacMahon Copas A.N., McComish S.F., Fletcher J.M., Caldwell M.A. (2021). The pathogenesis of parkinson’s disease: A complex interplay between astrocytes, microglia, and t lymphocytes?. Front. Neurol..

[7] Tolosa E., Garrido A., Scholz S.W., Poewe W. (2021). Challenges in the diagnosis of parkinson’s disease. Lancet Neurol..

[8] Dauer W., Przedborski S. (2003). Parkinson’s disease: Mechanisms and models. Neuron.

[9] Johnston J., Cushing L. (2020). Chemical exposures, health, and environmental justice in communities living on the fenceline of industry. Curr. Environ. Health Rep..

[10] US EPA (2023). Summary of the Toxic Substances Control Act.

[11] Fitzgerald J.A., Könemann S., Krümpelmann L., Županič A., Vom Berg C. (2021). Approaches to test the neurotoxicity of environmental contaminants in the zebrafish model: From behavior to molecular mechanisms. Environ. Toxicol. Chem..

[12] Weichbrod R.H., Thompson G.A., Norton J.N. (2018). Management of Animal Care and Use Programs in Research, Education, and Testing.

[13] Wolfle T.L. (2003). 50 years of the institute for laboratory animal research (ilar): 1953–2003. ILAR J..

[14] OECD (2008). Test No. 407: Repeated Dose 28-Day Oral Toxicity Study in Rodents.

[15] OECD (2018). Test No. 408: Repeated Dose 90-Day Oral Toxicity Study in Rodents.

[16] Smith A.J., Lilley E. (2019). The role of the three rs in improving the planning and reproducibility of animal experiments. Animals.

[17] (1986). Council Directive 86/609/eec of 24 November 1986 on the Approximation of Laws, Regulations and Administrative Provisions of the Member States Regarding the Protection of Animals Used for Experimental and Other Scientific Purposes. https://www.legislation.gov.uk/eudr/1986/609/contents.

[18] (2010). European Parliament (EP); Council of the European Union Directive 2010/63/eu on the protection of animals used for scientific purposes. EU Off. J..

[19] ECHA Echa and Alternatives to Animal Testing.

[20] Kang S.Y., Joshi P., Lee M.Y. (2021). High-throughput screening of compound neurotoxicity using 3d-cultured neural stem cells on a 384-pillar plate. Curr. Protoc..

[21] Shou W.Z. (2020). Current status and future directions of high-throughput adme screening in drug discovery. J. Pharm. Anal..

[22] Yoon M., Blaauboer B.J., Clewell H.J. (2015). Quantitative in vitro to in vivo extrapolation (qivive): An essential element for in vitro-based risk assessment. Toxicology.

[23] Wlodkowic D., Campana O. (2021). Toward high-throughput fish embryo toxicity tests in aquatic toxicology. Environ. Sci. Technol..

[24] OECD (2019). Test No. 203: Fish, Acute Toxicity Test.

[25] OECD (1998). Test No. 212: Fish, Short-Term Toxicity Test on Embryo and Sac-Fry Stages.

[26] OECD (2012). Test No. 305: Bioaccumulation in Fish: AQUEOUS and Dietary Exposure.

[27] OECD (2013). Test No. 210: Fish, Early-Life Stage Toxicity Test.

[28] Strähle U., Scholz S., Geisler R., Greiner P., Hollert H., Rastegar S., Schumacher A., Selderslaghs I., Weiss C., Witters H. (2012). Zebrafish embryos as an alternative to animal experiments—A commentary on the definition of the onset of protected life stages in animal welfare regulations. Reprod. Toxicol..

[29] Jarema K.A., Hunter D.L., Hill B.N., Olin J.K., Britton K.N., Waalkes M.R., Padilla S. (2022). Developmental neurotoxicity and behavioral screening in larval zebrafish with a comparison to other published results. Toxics.

[30] de Abreu M.S., Genario R., Giacomini A., Demin K.A., Lakstygal A.M., Amstislavskaya T.G., Fontana B.D., Parker M.O., Kalueff A.V. (2020). Zebrafish as a model of neurodevelopmental disorders. Neuroscience.

[31] d’Amora M., Giordani S. (2018). The utility of zebrafish as a model for screening developmental neurotoxicity. Front. Neurosci..

[32] Ali S., van Mil H.G., Richardson M.K. (2011). Large-scale assessment of the zebrafish embryo as a possible predictive model in toxicity testing. PLoS ONE.

[33] OECD (2013). Test No. 236: Fish Embryo Acute Toxicity (Fet) Test.

[34] Vaughan M., van Egmond R. (2010). The use of the zebrafish (*Danio rerio*) embryo for the acute toxicity testing of surfactants, as a possible alternative to the acute fish test. Altern. Lab. Anim..

[35] Ahkin Chin Tai J.K., Horzmann K.A., Franco J., Jannasch A.S., Cooper B.R., Freeman J.L. (2021). Developmental atrazine exposure in zebrafish produces the same major metabolites as mammals along with altered behavioral outcomes. Neurotoxicol. Teratol..

[36] Bailey J., Oliveri A., Levin E.D. (2013). Zebrafish model systems for developmental neurobehavioral toxicology. Birth Defects Res. C Embryo Today.

[37] Basnet R.M., Zizioli D., Taweedet S., Finazzi D., Memo M. (2019). Zebrafish larvae as a behavioral model in neuropharmacology. Biomedicines.

[38] Cassar S., Adatto I., Freeman J.L., Gamse J.T., Iturria I., Lawrence C., Muriana A., Peterson R.T., Van Cruchten S., Zon L.I. (2020). Use of zebrafish in drug discovery toxicology. Chem. Res. Toxicol..

[39] Goldsmith J.R., Jobin C. (2012). Think small: Zebrafish as a model system of human pathology. J. Biomed. Biotechnol..

[40] Bailone R.L., Fukushima H.C.S., Fernandes B.H.V., De Aguiar L.K., Corrêa T., Janke H., Setti P.G., Roça R.O., Borra R.C. (2020). Zebrafish as an alternative animal model in human and animal vaccination research. Lab. Anim. Res..

[41] Howe K., Clark M.D., Torroja C.F., Torrance J., Berthelot C., Muffato M., Collins J.E., Humphray S., McLaren K., Matthews L. (2013). The zebrafish reference genome sequence and its relationship to the human genome. Nature.

[42] Barbazuk W.B., Korf I., Kadavi C., Heyen J., Tate S., Wun E., Bedell J.A., McPherson J.D., Johnson S.L. (2000). The syntenic relationship of the zebrafish and human genomes. Genome Res..

[43] Kinkhabwala A., Riley M., Koyama M., Monen J., Satou C., Kimura Y., Higashijima S., Fetcho J. (2011). A structural and functional ground plan for neurons in the hindbrain of zebrafish. Proc. Natl. Acad. Sci. USA.

[44] Verma R., Choudhary P.R., Nirmal N.K., Syed F., Verma R. (2022). Neurotransmitter Systems in Zebrafish Model as a Target for Neurobehavioural Studies. Mater. Today Proc..

[45] Lal P., Kawakami K. (2022). Integrated behavioral, genetic and brain circuit visualization methods to unravel functional anatomy of zebrafish amygdala. Front. Neuroanat..

[46] Horzmann K.A., Freeman J.L. (2016). Zebrafish get connected: Investigating neurotransmission targets and alterations in chemical toxicity. Toxics.

[47] Sáez-Espinosa P., Franco-Esclapez C., Robles-Gómez L., Silva W., Romero A., Immler S., Gómez-Torres M.J. (2022). Morphological and ultrastructural alterations of zebrafish (*Danio rerio*) spermatozoa after motility activation. Theriogenology.

[48] Schmidt R., Strähle U., Scholpp S. (2013). Neurogenesis in zebrafish—From embryo to adult. Neural Dev..

[49] Woo K., Fraser S.E. (1995). Order and coherence in the fate map of the zebrafish nervous system. Development.

[50] Gutzman J.H., Graeden E.G., Lowery L.A., Holley H.S., Sive H. (2008). Formation of the zebrafish midbrain-hindbrain boundary constriction requires laminin-dependent basal constriction. Mech. Dev..

[51] Kimmel C.B., Ballard W.W., Kimmel S.R., Ullmann B., Schilling T.F. (1995). Stages of embryonic development of the zebrafish. Dev. Dyn..

[52] Carmean V., Ribera A.B. (2010). Genetic analysis of the touch response in zebrafish (*Danio rerio*). Int. J. Comp. Psychol..

[53] Chang J.T., Lehtinen M.K., Sive H. (2016). Zebrafish cerebrospinal fluid mediates cell survival through a retinoid signaling pathway. Dev. Neurobiol..

[54] Quiñonez-Silvero C., Hübner K., Herzog W. (2020). Development of the brain vasculature and the blood-brain barrier in zebrafish. Dev. Biol..

[55] Fleming A., Diekmann H., Goldsmith P. (2013). Functional characterisation of the maturation of the blood-brain barrier in larval zebrafish. PLoS ONE.

[56] Neely S.A., Lyons D.A. (2021). Insights into central nervous system glial cell formation and function from zebrafish. Front. Cell Dev. Biol..

[57] Bauer B., Mally A., Liedtke D. (2021). Zebrafish embryos and larvae as alternative animal models for toxicity testing. Int. J. Mol. Sci..

[58] Chu J., Sadler K.C. (2009). New school in liver development: Lessons from zebrafish. Hepatology.

[59] Sales Cadena M.R., Cadena P.G., Watson M.R., Sarmah S., Ii S.L.B., Marrs J.A. (2021). Zebrafish (*Danio rerio*) larvae show behavioral and embryonic development defects when exposed to opioids at embryo stage. Neurotoxicol. Teratol..

[60] Rosa J.G.S., Lima C., Lopes-Ferreira M. (2022). Zebrafish larvae behavior models as a tool for drug screenings and pre-clinical trials: A review. Int. J. Mol. Sci..

[61] McLean D.L., Fetcho J.R. (2004). Ontogeny and innervation patterns of dopaminergic, noradrenergic, and serotonergic neurons in larval zebrafish. J. Comp. Neurol..

[62] Wolf J.C., Segner H.E. (2023). Hazards of current concentration-setting practices in environmental toxicology studies. Crit. Rev. Toxicol..

[63] Gad S.C., Wexler P. (2014). Ld50/lc50 (lethal dosage 50/lethal concentration 50). Encyclopedia of Toxicology.

[64] Braunbeck T., Kais B., Lammer E., Otte J., Schneider K., Stengel D., Strecker R. (2015). The fish embryo test (fet): Origin, applications, and future. Environ. Sci. Pollut. Res. Int..

[65] Belanger S.E., Rawlings J.M., Carr G.J. (2013). Use of fish embryo toxicity tests for the prediction of acute fish toxicity to chemicals. Environ. Toxicol. Chem..

[66] Lammer E., Carr G.J., Wendler K., Rawlings J.M., Belanger S.E. (2009). Braunbeck. Is the fish embryo toxicity test (fet) with the zebrafish (*Danio rerio*) a potential alternative for the fish acute toxicity test?. Comp. Biochem. Physiol. C Toxicol. Pharmacol..

[67] Sobanska M., Scholz S., Nyman A.M., Cesnaitis R., Alonso S.G., Klüver N., Kühne R., Tyle H., de Knecht J., Dang Z. (2018). Applicability of the fish embryo acute toxicity (fet) test (oecd 236) in the regulatory context of registration, evaluation, authorisation, and restriction of chemicals (reach). Environ. Toxicol. Chem..

[68] Klüver N., König M., Ortmann J., Massei R., Paschke A., Kühne R., Scholz S. (2015). Fish embryo toxicity test: Identification of compounds with weak toxicity and analysis of behavioral effects to improve prediction of acute toxicity for neurotoxic compounds. Environ. Sci. Technol..

[69] Glaberman S., Padilla S., Barron M.G. (2017). Evaluating the zebrafish embryo toxicity test for pesticide hazard screening. Environ. Toxicol. Chem..

[70] Henn K., Braunbeck T. (2011). Dechorionation as a tool to improve the fish embryo toxicity test (fet) with the zebrafish (*Danio rerio*). Comp. Biochem. Physiol. C Toxicol. Pharmacol..

[71] Olivares C.I., Field J.A., Simonich M., Tanguay R.L., Sierra-Alvarez R. (2016). Arsenic (iii, v), indium (iii), and gallium (iii) toxicity to zebrafish embryos using a high-throughput multi-endpoint in vivo developmental and behavioral assay. Chemosphere.

[72] Coral J.A., Heaps S., Glaholt S.P., Karty J.A., Jacobson S.C., Shaw J.R., Bondesson M. (2021). Arsenic exposure induces a bimodal toxicity response in zebrafish. Environ. Pollut..

[73] Ball N., Teo W.P., Chandra S., Chapman J. (2019). Parkinson’s disease and the environment. Front. Neurol..

[74] Huat T.J., Camats-Perna J., Newcombe E.A., Valmas N., Kitazawa M., Medeiros R. (2019). Metal toxicity links to alzheimer’s disease and neuroinflammation. J. Mol. Biol..

[75] Rana S.V. (2008). Metals and apoptosis: Recent developments. J. Trace Elem. Med. Biol..

[76] Franco R., Sánchez-Olea R., Reyes-Reyes E.M., Panayiotidis M.I. (2009). Environmental toxicity, oxidative stress and apoptosis: Ménage à trois. Mutat. Res..

[77] Dionísio P.A., Amaral J.D., Rodrigues C.M.P. (2021). Oxidative stress and regulated cell death in parkinson’s disease. Ageing Res. Rev..

[78] Sayre L.M., Perry G., Smith M.A. (2008). Oxidative stress and neurotoxicity. Chem. Res. Toxicol..

[79] Chen Z., Zhong C. (2014). Oxidative stress in alzheimer’s disease. Neurosci. Bull..

[80] Goel P., Chakrabarti S., Goel K., Bhutani K., Chopra T., Bali S. (2022). Neuronal Cell Death Mechanisms in Alzheimer’s Disease: An Insight. Front. Mol. Neurosci..

[81] Zhao Y., Zhao B. (2013). Oxidative stress and the pathogenesis of alzheimer’s disease. Oxidative Med. Cell Longev..

[82] Glenner G.G., Wong C.W. (1984). Alzheimer’s disease: Initial report of the purification and characterization of a novel cerebrovascular amyloid protein. Biochem. Biophys. Res. Commun..

[83] Betteridge D.J. (2000). What is oxidative stress?. Metabolism.

[84] Tönnies E., Trushina E. (2017). Oxidative stress, synaptic dysfunction, and alzheimer’s disease. J. Alzheimers Dis..

[85] Wilkinson B.L., Landreth G.E. (2006). The microglial nadph oxidase complex as a source of oxidative stress in alzheimer’s disease. J. Neuroinflamm..

[86] Bisht K., Sharma K., Tremblay M.-È. (2018). Chronic Stress as a Risk Factor for Alzheimer’s Disease: Roles of Microglia-Mediated Synaptic Remodeling, Inflammation, and Oxidative Stress. Neurobiol. Stress.

[87] Exley C. (2014). What is the risk of aluminium as a neurotoxin?. Expert Rev. Neurother..

[88] Flaten T.P. (2001). Aluminium as a risk factor in alzheimer’s disease, with emphasis on drinking water. Brain Res. Bull..

[89] Martyn C.N., Barker D.J., Osmond C., Harris E.C., Edwardson J.A., Lacey R.F. (1989). Geographical relation between alzheimer’s disease and aluminum in drinking water. Lancet.

[90] Oshima E., Ishihara T., Yokota O., Nakashima-Yasuda H., Nagao S., Ikeda C., Naohara J., Terada S., Uchitomi Y. (2013). Accelerated tau aggregation, apoptosis and neurological dysfunction caused by chronic oral administration of aluminum in a mouse model of tauopathies. Brain Pathol..

[91] Kaur K., Narang R.K., Singh S. (2022). Alcl(3) induced learning and memory deficit in zebrafish. Neurotoxicology.

[92] Lee J., Peterson S.M., Freeman J.L. (2017). Sex-specific characterization and evaluation of the alzheimer’s disease genetic risk factor sorl1 in zebrafish during aging and in the adult brain following a 100 ppb embryonic lead exposure. J. Appl. Toxicol..

[93] Tanner C.M., Goldman S.M. (1996). Epidemiology of parkinson’s disease. Neurol. Clin..

[94] Hayes M.T. (2019). Parkinson’s disease and parkinsonism. Am. J. Med..

[95] Borghammer P. (2021). The α-synuclein origin and connectome model (soc model) of parkinson’s disease: Explaining motor asymmetry, non-motor phenotypes, and cognitive decline. J. Parkinsons Dis..

[96] Miller D.B., O’Callaghan J.P. (2015). Biomarkers of parkinson’s disease: Present and future. Metabolism.

[97] Emamzadeh F.N., Surguchov A. (2018). Parkinson’s disease: Biomarkers, treatment, and risk factors. Front. Neurosci..

[98] Hartmann A., Hunot S., Michel P.P., Muriel M.P., Vyas S., Faucheux B.A., Mouatt-Prigent A., Turmel H., Srinivasan A., Ruberg M. (2000). Caspase-3: A vulnerability factor and final effector in apoptotic death of dopaminergic neurons in parkinson’s disease. Proc. Natl. Acad. Sci. USA.

[99] Hartmann A., Troadec J.D., Hunot S., Kikly K., Faucheux B.A., Mouatt-Prigent A., Ruberg M., Agid Y., Hirsch E.C. (2001). Caspase-8 is an effector in apoptotic death of dopaminergic neurons in parkinson’s disease, but pathway inhibition results in neuronal necrosis. J. Neurosci..

[100] Yuan H., Zheng J.C., Liu P., Zhang S.F., Xu J.Y., Bai L.M. (2007). Pathogenesis of parkinson’s disease: Oxidative stress, environmental impact factors and inflammatory processes. Neurosci. Bull..

[101] Heinz S., Freyberger A., Lawrenz B., Schladt L., Schmuck G., Ellinger-Ziegelbauer H. (2017). Mechanistic investigations of the mitochondrial complex i inhibitor rotenone in the context of pharmacological and safety evaluation. Sci. Rep..

[102] Liu M., Choi D.Y., Hunter R.L., Pandya J.D., Cass W.A., Sullivan P.G., Kim H.C., Gash D.M., Bing G. (2010). Trichloroethylene induces dopaminergic neurodegeneration in fisher 344 rats. J. Neurochem..

[103] Wang X.H., Souders C.L., Zhao Y.H., Martyniuk C.J. (2018). Paraquat affects mitochondrial bioenergetics, dopamine system expression, and locomotor activity in zebrafish (*Danio rerio*). Chemosphere.

[104] Liu X., Zhang R., Jin Y. (2020). Differential responses of larval zebrafish to the fungicide propamocarb: Endpoints at development, locomotor behavior and oxidative stress. Sci. Total Environ..

[105] Kalyn M., Lee H., Curry J., Tu W., Ekker M., Mennigen J.A. (2023). Effects of pfos, f-53b and obs on Locomotor Behaviour, the Dopaminergic System and Mitochondrial Function in Developing Zebrafish (*Danio rerio*). Environ. Pollut..

[106] Alfalahi H., Dias S.B., Khandoker A.H., Chaudhuri K.R., Hadjileontiadis L.J. (2023). A scoping review of neurodegenerative manifestations in explainable digital phenotyping. NPJ Parkinsons Dis..

[107] Birben E., Sahiner U.M., Sackesen C., Erzurum S., Kalayci O. (2012). Oxidative stress and antioxidant defense. World Allergy Organ. J..

[108] Murphy M.P., Bayir H., Belousov V., Chang C.J., Davies K.J.A., Davies M.J., Dick T.P., Finkel T., Forman H.J., Janssen-Heininger Y. (2022). Guidelines for measuring reactive oxygen species and oxidative damage in cells and in vivo. Nat. Metab..

[109] Valko M., Morris H., Cronin M.T. (2005). Metals, toxicity and oxidative stress. Curr. Med. Chem..

[110] Cobley J.N., Fiorello M.L., Bailey D.M. (2018). 13 reasons why the brain is susceptible to oxidative stress. Redox Biol..

[111] Percário S., da Silva Barbosa A., Varela E.L.P., Gomes A.R.Q., Ferreira M.E.S., de Nazaré Araújo Moreira T., Dolabela M.F. (2020). Oxidative stress in parkinson’s disease: Potential benefits of antioxidant supplementation. Oxidative Med. Cell Longev..

[112] Ni A., Ernst C. (2022). Evidence that substantia nigra pars compacta dopaminergic neurons are selectively vulnerable to oxidative stress because they are highly metabolically active. Front. Cell Neurosci..

[113] Abbate F., Maugeri A., Laurà R., Levanti M., Navarra M., Cirmi S., Germanà A. (2021). Zebrafish as a useful model to study oxidative stress-linked disorders: Focus on flavonoids. Antioxidants.

[114] Somogyi A., Rosta K., Pusztai P., Tulassay Z., Nagy G. (2007). Antioxidant measurements. Physiol. Meas..

[115] Kim H., Xue X. (2020). Detection of total reactive oxygen species in adherent cells by 2′,7′-dichlorodihydrofluorescein diacetate staining. J. Vis. Exp..

[116] Katerji M., Filippova M., Duerksen-Hughes P. (2019). Approaches and methods to measure oxidative stress in clinical samples: Research applications in the cancer field. Oxidative Med. Cell Longev..

[117] Bode K., Link C., Krammer P.H., Weyd H. (2020). Flow-cytometric detection of low-level reactive oxygen species in cell lines and primary immune cells. Bio Protoc..

[118] Ng N.S., Ooi L. (2021). A simple microplate assay for reactive oxygen species generation and rapid cellular protein normalization. Bio Protoc..

[119] Yang D., Lauridsen H., Buels K., Chi L.H., La Du J., Bruun D.A., Olson J.R., Tanguay R.L., Lein P.J. (2011). Chlorpyrifos-oxon disrupts zebrafish axonal growth and motor behavior. Toxicol. Sci..

[120] Valavanidis A., Vlachogianni T., Fiotakis C. (2009). 8-hydroxy-2′-deoxyguanosine (8-ohdg): A critical biomarker of oxidative stress and carcinogenesis. J. Environ. Sci. Health C Environ. Carcinog. Ecotoxicol. Rev..

[121] Urbaniak S.K., Boguszewska K., Szewczuk M., Kaźmierczak-Barańska J., Karwowski B.T. (2020). 8-oxo-7,8-dihydro-2′-deoxyguanosine (8-oxodg) and 8-hydroxy-2′-deoxyguanosine (8-ohdg) as a potential biomarker for gestational diabetes mellitus (gdm) development. Molecules.

[122] McKelvey-Martin V.J., Green M.H., Schmezer P., Pool-Zobel B.L., De Méo M.P., Collins A. (1993). The single cell gel electrophoresis assay (comet assay): A european review. Mutat. Res..

[123] Ayala A., Muñoz M.F., Argüelles S. (2014). Lipid peroxidation: Production, metabolism, and signaling mechanisms of malondialdehyde and 4-hydroxy-2-nonenal. Oxidative Med. Cell Longev..

[124] Yoshida Y., Umeno A., Shichiri M. (2013). Lipid peroxidation biomarkers for evaluating oxidative stress and assessing antioxidant capacity in vivo. J. Clin. Biochem. Nutr..

[125] Aguilar Diaz De Leon J., Borges C.R. (2020). Evaluation of oxidative stress in biological samples using the thiobarbituric acid reactive substances assay. J. Vis. Exp..

[126] Apak R. (2019). Current issues in antioxidant measurement. J. Agric. Food Chem..

[127] Zhu B., He W., Hu S., Kong R., Yang L. (2019). The fate and oxidative stress of different sized sio(2) nanoparticles in zebrafish (*Danio rerio*) larvae. Chemosphere.

[128] Guru A., Arockiaraj J. (2023). Exposure to environmental pollutant bisphenol a causes oxidative damage and lipid accumulation in zebrafish larvae: Protective role of wl15 peptide derived from cysteine and glycine-rich protein 2. J. Biochem. Mol. Toxicol..

[129] Baran A., Yildirim S., Ghosigharehaghaji A., Bolat İ., Sulukan E., Ceyhun S.B. (2021). An approach to evaluating the potential teratogenic and neurotoxic mechanism of bha based on apoptosis induced by oxidative stress in zebrafish embryo (*Danio rerio*). Hum. Exp. Toxicol..

[130] Spitz D.R., Oberley L.W. (1989). An assay for superoxide dismutase activity in mammalian tissue homogenates. Anal. Biochem..

[131] Zitka O., Skalickova S., Gumulec J., Masarik M., Adam V., Hubalek J., Trnkova L., Kruseova J. (2012). Eckschlager and R. Kizek. Redox status expressed as gsh:Gssg ratio as a marker for oxidative stress in paediatric tumour patients. Oncol. Lett..

[132] Beers R.F., Sizer I.W. (1952). A spectrophotometric method for measuring the breakdown of hydrogen peroxide by catalase. J. Biol. Chem..

[133] Sun H.J., Zhang J.Y., Wang Q., Zhu E., Chen W., Lin H., Chen J., Hong H. (2019). Environmentally relevant concentrations of arsenite induces developmental toxicity and oxidative responses in the early life stage of zebrafish. Environ. Pollut..

[134] Adeyemi J.A., da Cunha Martins-Junior A., Barbosa F. (2015). Teratogenicity, genotoxicity and oxidative stress in zebrafish embryos (*Danio rerio*) co-exposed to arsenic and atrazine. Comp. Biochem. Physiol. C Toxicol. Pharmacol..

[135] Liu H., Gooneratne R., Huang X., Lai R., Wei J., Wang W. (2015). A rapid in vivo zebrafish model to elucidate oxidative stress-mediated pcb126-induced apoptosis and developmental toxicity. Free Radic. Biol. Med..

[136] Kusik B.W., Carvan M.J., Udvadia A.J. (2008). Detection of mercury in aquatic environments using epre reporter zebrafish. Mar. Biotechnol..

[137] Ngo V., Duennwald M.L. (2022). Nrf2 and oxidative stress: A general overview of mechanisms and implications in human disease. Antioxidants.

[138] Mourabit S., Fitzgerald J.A., Ellis R.P., Takesono A., Porteus C.S., Trznadel M., Metz J., Winter M.J., Kudoh T., Tyler C.R. (2019). New insights into organ-specific oxidative stress mechanisms using a novel biosensor zebrafish. Environ. Int..

[139] Klim J., Gładki A., Kucharczyk R., Zielenkiewicz U., Kaczanowski S. (2018). Ancestral state reconstruction of the apoptosis machinery in the common ancestor of eukaryotes. G3.

[140] Fadeel B., Orrenius S. (2005). Apoptosis: A basic biological phenomenon with wide-ranging implications in human disease. J. Intern. Med..

[141] Elmore S. (2007). Apoptosis: A review of programmed cell death. Toxicol. Pathol..

[142] D’Arcy M.S. (2019). Cell death: A review of the major forms of apoptosis, necrosis and autophagy. Cell Biol. Int..

[143] McGrath P., Seng W.L. (2013). Use of zebrafish apoptosis assays for preclinical drug discovery. Expert Opin. Drug Discov..

[144] Sorrells S., Toruno C., Stewart R.A., Jette C. (2013). Analysis of apoptosis in zebrafish embryos by whole-mount immunofluorescence to detect activated caspase 3. J. Vis. Exp..

[145] Tucker B., Lardelli M. (2007). A rapid apoptosis assay measuring relative acridine orange fluorescence in zebrafish embryos. Zebrafish.

[146] Banfalvi G. (2017). Methods to detect apoptotic cell death. Apoptosis.

[147] Butterick T.A., Duffy C.M., Lee R.E., Billington C.J., Kotz C.M., Nixon J.P. (2014). Use of a caspase multiplexing assay to determine apoptosis in a hypothalamic cell model. J. Vis. Exp..

[148] Kyrylkova K., Kyryachenko S., Leid M., Kioussi C. (2012). Detection of apoptosis by tunel assay. Methods Mol. Biol..

[149] Majtnerová P., Roušar T. (2018). An overview of apoptosis assays detecting DNA fragmentation. Mol. Biol. Rep..

[150] van Engeland M., Nieland L.J., Ramaekers F.C., Schutte B., Reutelingsperger C.P. (1998). Annexin v-affinity assay: A review on an apoptosis detection system based on phosphatidylserine exposure. Cytometry.

[151] Crowley L.C., Marfell B.J., Waterhouse N.J. (2016). Analyzing cell death by nuclear staining with hoechst 33342. Cold Spring Harb. Protoc..

[152] Frankfurt O.S., Robb J.A., Sugarbaker E.V., Villa L. (1996). Monoclonal antibody to single-stranded DNA is a specific and sensitive cellular marker of apoptosis. Exp. Cell Res..

[153] Ribble D., Goldstein N.B., Norris D.A., Shellman Y.G. (2005). A simple technique for quantifying apoptosis in 96-well plates. BMC Biotechnol..

[154] Strugger S., Degode F. (1941). Fluoreszenzmikroskopische untersuchungen über die aufnahme und speicherung des akridinorange durch lebende und tote pflanzenzellen. Protoplasma.

[155] Pinheiro-da-Silva J., Luchiari A.C. (2021). Embryonic ethanol exposure on zebrafish early development. Brain Behav..

[156] Parlak V. (2018). Evaluation of apoptosis, oxidative stress responses, ache activity and body malformations in zebrafish (*Danio rerio*) embryos exposed to deltamethrin. Chemosphere.

[157] Zhu J., Wang C., Gao X., Wang L., Cao S., Wu Q., Qiao S., Zhang Z., Li L. (2019). Comparative effects of mercury chloride and methylmercury exposure on early neurodevelopment in zebrafish larvae. RSC Adv..

[158] Monaco A., Capriello T., Grimaldi M.C., Schiano V., Ferrandino I. (2017). Neurodegeneration in zebrafish embryos and adults after cadmium exposure. Eur. J. Histochem..

[159] Mirzayans R., Murray D. (2020). Do tunel and other apoptosis assays detect cell death in preclinical studies?. Int. J. Mol. Sci..

[160] Guo M., Lu B., Gan J., Wang S., Jiang X., Li H. (2021). Apoptosis detection: A purpose-dependent approach selection. Cell Cycle.

[161] van Ham T.J., Mapes J., Kokel D., Peterson R.T. (2010). Live imaging of apoptotic cells in zebrafish. FASEB J..

[162] Yamashita M., Mizusawa N., Hojo M., Yabu T. (2008). Extensive apoptosis and abnormal morphogenesis in pro-caspase-3 transgenic zebrafish during development. J. Exp. Biol..

[163] Mirsaeidi M., Gidfar S., Vu A., Schraufnagel D. (2016). Annexins family: Insights into their functions and potential role in pathogenesis of sarcoidosis. J. Transl. Med..

[164] Farber S.A., De Rose R.A., Olson E.S., Halpern M.E. (2003). The zebrafish annexin gene family. Genome Res..

[165] Hyman S.E. (2005). Neurotransmitters. Curr. Biol..

[166] Teleanu R.I., Niculescu A.G., Roza E., Vladâcenco O., Grumezescu A.M., Teleanu D.M. (2022). Neurotransmitters-key factors in neurological and neurodegenerative disorders of the central nervous system. Int. J. Mol. Sci..

[167] Carvajal-Oliveros A., Campusano J.M. (2020). Studying the contribution of serotonin to neurodevelopmental disorders. Can this fly?. Front. Behav. Neurosci..

[168] Ncube D., Tallafuss A., Serafin J., Bruckner J., Farnsworth D.R., Miller A.C., Eisen J.S., Washbourne P. (2022). A conserved transcriptional fingerprint of multi-neurotransmitter neurons necessary for social behavior. BMC Genom..

[169] Li Y., Sun H., Chen Z., Xu H., Bu G., Zheng H. (2016). Implications of gabaergic neurotransmission in alzheimer’s disease. Front. Aging Neurosci..

[170] Fuertes I., Barata C. (2021). Characterization of neurotransmitters and related metabolites in daphnia magna juveniles deficient in serotonin and exposed to neuroactive chemicals that affect its behavior: A targeted lc-ms/ms method. Chemosphere.

[171] Pal M.M. (2021). Glutamate: The master neurotransmitter and its implications in chronic stress and mood disorders. Front. Hum. Neurosci..

[172] Meldrum B.S. (2000). Glutamate as a neurotransmitter in the brain: Review of physiology and pathology. J. Nutr..

[173] Reddy-Thootkur M., Kraguljac N.V., Lahti A.C. (2022). The role of glutamate and gaba in cognitive dysfunction in schizophrenia and mood disorders—A systematic review of magnetic resonance spectroscopy studies. Schizophr. Res..

[174] Owens D.F., Kriegstein A.R. (2002). Is there more to gaba than synaptic inhibition?. Nat. Rev. Neurosci..

[175] Ngo D.H., Vo T.S. (2019). An updated review on pharmaceutical properties of gamma-aminobutyric acid. Molecules.

[176] Shrivastava A.N., Triller A., Sieghart W. (2011). Gaba(a) receptors: Post-synaptic co-localization and cross-talk with other receptors. Front. Cell Neurosci..

[177] Gerrard L.B., Tantirigama M.L.S., Bekkers J.M. (2018). Pre- and postsynaptic activation of gaba(b) receptors modulates principal cell excitation in the piriform cortex. Front. Cell Neurosci..

[178] Chiapponi C., Piras F., Caltagirone C., Spalletta G. (2016). Gaba system in schizophrenia and mood disorders: A mini review on third-generation imaging studies. Front. Psychiatry.

[179] Schür R.R., Draisma L.W., Wijnen J.P., Boks M.P., Koevoets M.G., Joëls M., Klomp D.W., Kahn R.S., Vinkers C.H. (2016). Brain gaba levels across psychiatric disorders: A systematic literature review and meta-analysis of (1) h-mrs studies. Hum. Brain Mapp..

[180] Sarawagi A., Soni N.D., Patel A.B. (2021). Glutamate and gaba homeostasis and neurometabolism in major depressive disorder. Front. Psychiatry.

[181] Jung-Klawitter S., Hübschmann O.K. (2019). Analysis of catecholamines and pterins in inborn errors of monoamine neurotransmitter metabolism-from past to future. Cells.

[182] Provensi G., Costa A., Izquierdo I., Blandina P., Passani M.B. (2020). Brain histamine modulates recognition memory: Possible implications in major cognitive disorders. Br. J. Pharmacol..

[183] Passani M.B., Bacciottini L., Mannaioni P.F., Blandina P. (2000). Central histaminergic system and cognition. Neurosci. Biobehav. Rev..

[184] Rozov S.V., Porkka-Heiskanen T., Panula P. (2015). On the role of histamine receptors in the regulation of circadian rhythms. PLoS ONE.

[185] Ishizuka T., Nomura S., Hosoda H., Kangawa K., Watanabe T., Yamatodani A. (2006). A role of the histaminergic system for the control of feeding by orexigenic peptides. Physiol. Behav..

[186] Martinowich K., Lu B. (2008). Interaction between bdnf and serotonin: Role in mood disorders. Neuropsychopharmacology.

[187] Bao A.M., Swaab D.F. (2019). The human hypothalamus in mood disorders: The hpa axis in the center. IBRO Rep..

[188] Chaouloff F. (2000). Serotonin, stress and corticoids. J. Psychopharmacol..

[189] Rico E.P., Rosemberg D.B., Seibt K.J., Capiotti K.M., Da Silva R.S., Bonan C.D. (2011). Zebrafish neurotransmitter systems as potential pharmacological and toxicological targets. Neurotoxicol. Teratol..

[190] Cifuentes Castro V.H., Valenzuela C.L.L., Sánchez J.C.S., Peña K.P., Pérez S.J.L., Ibarra J.O., Villagrán A.M. (2014). An update of the classical and novel methods used for measuring fast neurotransmitters during normal and brain altered function. Curr. Neuropharmacol..

[191] Tufi S., Leonards P., Lamoree M., de Boer J., Legler J., Legradi J. (2016). Changes in neurotransmitter profiles during early zebrafish (*Danio rerio*) development and after pesticide exposure. Environ. Sci. Technol..

[192] Wirbisky S.E., Weber G.J., Sepúlveda M.S., Xiao C., Cannon J.R., Freeman J.L. (2015). Developmental origins of neurotransmitter and transcriptome alterations in adult female zebrafish exposed to atrazine during embryogenesis. Toxicology.

[193] Weber G.J., Sepúlveda M.S., Peterson S.M., Lewis S.S., Freeman J.L. (2013). Transcriptome alterations following developmental atrazine exposure in zebrafish are associated with disruption of neuroendocrine and reproductive system function, cell cycle, and carcinogenesis. Toxicol. Sci..

[194] Wirbisky S.E., Weber G.J., Lee J.W., Cannon J.R., Freeman J.L. (2014). Novel dose-dependent alterations in excitatory gaba during embryonic development associated with lead (pb) neurotoxicity. Toxicol. Lett..

[195] Kanungo J., Twaddle N.C., Silva C., Robinson B., Wolle M., Conklin S., MacMahon S., Gu Q., Edhlund I., Benjamin L. (2023). Inorganic arsenic alters the development of dopaminergic neurons but not serotonergic neurons and induces motor neuron development via sonic hedgehog pathway in zebrafish. Neurosci. Lett..

[196] Ding P., Xiang C., Li X., Chen H., Shi X., Huang C., Yu Y., Qi J., Li A.J., Zhang L. (2023). Photoaged microplastics induce neurotoxicity via oxidative stress and abnormal neurotransmission in zebrafish larvae (*Danio rerio*). Sci. Total Environ..

[197] Nikolac Perkovic M., Paska A.V., Konjevod M., Kouter K., Strac D.S., Erjavec G.N., Pivac N. (2021). Epigenetics of alzheimer’s disease. Biomolecules.

[198] Almatarneh M.H., Kayed G.G., Abbad S.S.A., Alsunaidi Z.H.A., Al-Sheraideh M.S., Zhao Y. (2022). Mechanistic Study on DNA Mutation of the Cytosine Methylation Reaction at c5 Position. Arab. J. Chem..

[199] Elhamamsy A.R. (2017). Role of DNA Methylation in Imprinting Disorders: An Updated Review. J. Assist. Reprod. Genet..

[200] Jin B., Robertson K.D. (2013). DNA Methyltransferases, DNA Damage Repair, and Cancer. Adv. Exp. Med. Biol..

[201] Millán-Zambrano G., Burton A., Bannister A.J., Schneider R. (2022). Histone post-translational modifications—Cause and consequence of genome function. Nat. Rev. Genet..

[202] Bannister A.J., Kouzarides T. (2011). Regulation of chromatin by histone modifications. Cell Res..

[203] Santana D.A., Smith M.A.C., Chen E.S. (2023). Histone modifications in alzheimer’s disease. Genes.

[204] Morris K.V. (2009). Non-Coding RNAs, Epigenetic Memory and the Passage of Information to Progeny. RNA Biol..

[205] Kaikkonen M.U., Lam M.T., Glass C.K. (2011). Non-Coding RNAs as Regulators of Gene Expression and Epigenetics. Cardiovasc. Res..

[206] Cavalieri V., Spinelli G. (2017). Environmental epigenetics in zebrafish. Epigenetics Chromatin.

[207] Li Y., Park C., Li X. (2018). Base modifications: Regulation of stem cell functions and diseases. Stem Cells Int..

[208] Nomura W. (2018). Development of toolboxes for precision genome/epigenome editing and imaging of epigenetics. Chem. Rec..

[209] Waryah C.B., Moses C., Arooj M., Blancafort P. (2018). Zinc fingers, tales, and crispr systems: A comparison of tools for epigenome editing. Methods Mol. Biol..

[210] Balasubramanian S., Raghunath A., Perumal E. (2019). Role of epigenetics in zebrafish development. Gene.

[211] Kamstra J.H., Aleström P., Kooter J.M., Legler J. (2015). Zebrafish as a model to study the role of DNA methylation in environmental toxicology. Environ. Sci. Pollut. Res. Int..

[212] Li Y. (2021). Modern epigenetics methods in biological research. Methods.

[213] Miyata K., Naito M., Miyata T., Mokuda S., Asahara H. (2017). Bisulfite sequencing for DNA methylation analysis of primary muscle stem cells. Methods Mol. Biol..

[214] Li Y., Tollefsbol T.O. (2011). DNA methylation detection: Bisulfite genomic sequencing analysis. Methods Mol. Biol..

[215] Strömqvist M., Tooke N., Brunström B. (2010). DNA methylation levels in the 5′ flanking region of the vitellogenin i gene in liver and brain of adult zebrafish (*Danio rerio*)—Sex and tissue differences and effects of 17alpha-ethinylestradiol exposure. Aquat. Toxicol..

[216] Martin C.C., Laforest L., Akimenko M.A., Ekker M. (1999). A role for DNA methylation in gastrulation and somite patterning. Dev. Biol..

[217] Macleod D., Clark V.H., Bird A. (1999). Absence of genome-wide changes in DNA methylation during development of the zebrafish. Nat. Genet..

[218] Searle B., Müller M., Carell T., Kellett A. (2023). Third-generation sequencing of epigenetic DNA. Angew. Chem..

[219] Yan H., Tian S., Slager S.L., Sun Z. (2016). Chip-seq in studying epigenetic mechanisms of disease and promoting precision medicine: Progresses and future directions. Epigenomics.

[220] Bogdanović O., Fernández-Miñán A., Tena J.J., de la Calle-Mustienes E., Gómez-Skarmeta J.L. (2013). The developmental epigenomics toolbox: Chip-seq and methylcap-seq profiling of early zebrafish embryos. Methods.

[221] Terrazas-Salgado L., García-Gasca A., Betancourt-Lozano M., Llera-Herrera R., Alvarado-Cruz I., Yáñez-Rivera B. (2022). Epigenetic transgenerational modifications induced by xenobiotic exposure in zebrafish. Front. Cell Dev. Biol..

[222] O’Neill L.P., VerMilyea M.D., Turner B.M. (2006). Epigenetic characterization of the early embryo with a chromatin immunoprecipitation protocol applicable to small cell populations. Nat. Genet..

[223] Wang S., Bryan C., Xie J., Zhao H., Lin L.F., Tai J.A.C., Horzmann K.A., Sanchez O.F., Zhang M., Freeman J.L. (2022). Atrazine exposure in zebrafish induces aberrant genome-wide methylation. Neurotoxicol. Teratol..

[224] Lakstygal A.M., de Abreu M.S., Kalueff A.V. (2018). Zebrafish models of epigenetic regulation of cns functions. Brain Res. Bull..

[225] Bian X., Gao Y. (2021). DNA methylation and gene expression alterations in zebrafish embryos exposed to cadmium. Environ. Sci. Pollut. Res. Int..

[226] Wirbisky S.E., Weber G.J., Schlotman K.E., Sepúlveda M.S., Freeman J.L. (2016). Embryonic atrazine exposure alters zebrafish and human mirnas associated with angiogenesis, cancer, and neurodevelopment. Food Chem. Toxicol..

[227] Anger W.K. (2003). Neurobehavioural tests and systems to assess neurotoxic exposures in the workplace and community. Occup. Environ. Med..

[228] Kalueff A.V., Gebhardt M., Stewart A.M., Cachat J.M., Brimmer M., Chawla J.S., Craddock C., Kyzar E.J., Roth A., Landsman S. (2013). Towards a comprehensive catalog of zebrafish behavior 1.0 and beyond. Zebrafish.

[229] Vorhees C.V., Williams M.T., Hawkey A.B., Levin E.D. (2021). Translating neurobehavioral toxicity across species from zebrafish to rats to humans: Implications for risk assessment. Front. Toxicol..

[230] Stewart A., Gaikwad S., Kyzar E., Green J., Roth A., Kalueff A.V. (2012). Modeling anxiety using adult zebrafish: A conceptual review. Neuropharmacology.

[231] Menelaou E., Husbands E.E., Pollet R.G., Coutts C.A., Ali D.W., Svoboda K.R. (2008). Embryonic motor activity and implications for regulating motoneuron axonal pathfinding in zebrafish. Eur. J. Neurosci..

[232] Saint-Amant L., Drapeau P. (1998). Time course of the development of motor behaviors in the zebrafish embryo. J. Neurobiol..

[233] McKeown K.A., Downes G.B., Hutson L.D. (2009). Modular laboratory exercises to analyze the development of zebrafish motor behavior. Zebrafish.

[234] McClenahan P., Troup M., Scott E.K. (2012). Fin-tail coordination during escape and predatory behavior in larval zebrafish. PLoS ONE.

[235] Winata C.L., Korzh S., Kondrychyn I., Zheng W., Korzh V., Gong Z. (2009). Development of Zebrafish Swimbladder: The Requirement of Hedgehog Signaling in Specification and Organization of the Three Tissue Layers. Dev. Biol..

[236] Schnörr S.J., Steenbergen P.J., Richardson M.K., Champagne D.L. (2012). Measuring thigmotaxis in larval zebrafish. Behav. Brain Res..

[237] Carbaugh C.M., Widder M.W., Phillips C.S., Jackson D.A., DiVito V.T., van der Schalie W.H., Glover K.P. (2020). Assessment of zebrafish embryo photomotor response sensitivity and phase-specific patterns following acute- and long-duration exposure to neurotoxic chemicals and chemical weapon precursors. J. Appl. Toxicol..

[238] Fleisch V.C., Neuhauss S.C. (2006). Visual behavior in zebrafish. Zebrafish.

[239] Horzmann K.A., Freeman J.L. (2018). Making waves: New developments in toxicology with the zebrafish. Toxicol. Sci..

[240] Ortmann J., Altenburger R., Scholz S., Luckenbach T. (2022). Photomotor response data analysis approach to assess chemical neurotoxicity with the zebrafish embryo. Altex.

[241] Reif D.M., Truong L., Mandrell D., Marvel S., Zhang G., Tanguay R.L. (2016). High-throughput characterization of chemical-associated embryonic behavioral changes predicts teratogenic outcomes. Arch. Toxicol..

[242] Kokel D., Dunn T.W., Ahrens M.B., Alshut R., Cheung C.Y., Saint-Amant L., Bruni G., Mateus R., van Ham T.J., Shiraki T. (2013). Identification of nonvisual photomotor response cells in the vertebrate hindbrain. J. Neurosci..

[243] Kokel D., Peterson R.T. (2011). Using the zebrafish photomotor response for psychotropic drug screening. Methods Cell Biol..

[244] Burton C.E., Zhou Y., Bai Q., Burton E.A. (2017). Spectral properties of the zebrafish visual motor response. Neurosci. Lett..

[245] Horzmann K.A., Portales A.M., Batcho K.G., Freeman J.L. (2020). Developmental toxicity of trichloroethylene in zebrafish (*Danio rerio*). Environ. Sci. Process Impacts.

[246] Huang M., Ivantsova E., Konig I., Patel N., English C., Souders C.L., Martyniuk C.J. (2023). Developmental and mitochondrial toxicity assessment of perfluoroheptanoic acid (pfhpa) in zebrafish (*Danio rerio*). Environ. Toxicol. Pharmacol..

[247] Petrovic M., Farré M., Eljarrat E., Diaz S., Barceló D., Fanali S., Haddad P.R., Poole C.F., Schoenmakers P., Lloyd D. (2013). Chapter 14—Environmental analysis: Emerging pollutants. Liquid Chromatography.

[248] Gyimah E., Zhu X., Zhang Z., Guo M., Xu H., Mensah J.K., Dong X., Gyimah G.N.W. (2022). Oxidative stress and apoptosis in bisphenol af-induced neurotoxicity in zebrafish embryos. Environ. Toxicol. Chem..

[249] Lockwood B., Bjerke S., Kobayashi K., Guo S. (2004). Acute effects of alcohol on larval zebrafish: A genetic system for large-scale screening. Pharmacol. Biochem. Behav..

[250] Li F., Lin J., Liu X., Li W., Ding Y., Zhang Y., Zhou S., Guo N., Li Q. (2018). Characterization of the Locomotor Activities of Zebrafish Larvae under the Influence of Various Neuroactive Drugs. Ann. Transl. Med..

[251] Norton W., Bally-Cuif L. (2010). Adult zebrafish as a model organism for behavioural genetics. BMC Neurosci..

[252] Kenney J.W., Gerlai R.T. (2020). Chapter 12—Associative and nonassociative learning in adult zebrafish. Behavioral and Neural Genetics of Zebrafish.

[253] Tan J.K., Nazar F.H., Makpol S., Teoh S.L. (2022). Zebrafish: A pharmacological model for learning and memory research. Molecules.

[254] Cachat J.M., Canavello P.R., Elkhayat S.I., Bartels B.K., Hart P.C., Elegante M.F., Beeson E.C., Laffoon A.L., Haymore W.A.M., Tien D.H., Kalueff A.V., Cachat J.M. (2011). Video-aided analysis of zebrafish locomotion and anxiety-related behavioral responses. Zebrafish Neurobehavioral Protocols.

[255] Wang J., Wang D., Hu G., Yang L., Yan D., Wang M., Serikuly N., Alpyshov E., Amstislavskaya T.G., Demin K.A. (2020). A new method for vibration-based neurophenotyping of zebrafish. J. Neurosci. Methods.

[256] Stewart A.M., Gaikwad S., Kyzar E., Kalueff A.V. (2012). Understanding spatio-temporal strategies of adult zebrafish exploration in the open field test. Brain Res..

[257] Ahmad F., Richardson M.K. (2013). Exploratory behaviour in the open field test adapted for larval zebrafish: Impact of environmental complexity. Behav. Process..

[258] Benvenutti R., Marcon M., Gallas-Lopes M., de Mello A.J., Herrmann A.P., Piato A. (2021). Swimming in the Maze: An Overview of Maze Apparatuses and Protocols to Assess Zebrafish Behavior. Neurosci. Biobehav. Rev..

[259] Mathur P., Guo S. (2011). Differences of acute versus chronic ethanol exposure on anxiety-like behavioral responses in zebrafish. Behav. Brain Res..

[260] Wang H., Mu S., Zhang F., Liu H., Zhang H., Kang X. (2015). Effects of Atrazine on the Development of Neural System of Zebrafish, Danio rerio. Biomed. Res. Int..

[261] Glazer L., Brennan C.H. (2021). Developmental exposure to low concentrations of methylmercury causes increase in anxiety-related behaviour and locomotor impairments in zebrafish. Int. J. Mol. Sci..

[262] Bloch S., Thomas M., Colin I., Galant S., Machado E., Affaticati P., Jenett A., Yamamoto K. (2019). Mesencephalic origin of the inferior lobe in zebrafish. BMC Biol..

[263] Bridges C.C., Krasnikov B.F., Joshee L., Pinto J.T., Hallen A., Li J., Zalups R.K., Cooper A.J. (2012). New insights into the metabolism of organomercury compounds: Mercury-containing cysteine s-conjugates are substrates of human glutamine transaminase k and potent inactivators of cystathionine γ-lyase. Arch. Biochem. Biophys..

[264] Henriques M.C., Carvalho I., Santos C., Herdeiro M.T., Fardilha M., Pavlaki M.D., Loureiro S. (2023). Unveiling the molecular mechanisms and developmental consequences of mercury (hg) toxicity in zebrafish embryo-larvae: A comprehensive approach. Neurotoxicol. Teratol..

[265] Sun X., Yang Q., Jing M., Jia X., Tian L., Tao J. (2023). Environmentally relevant concentrations of organic (benzophenone-3) and inorganic (titanium dioxide nanoparticles) uv filters co-exposure induced neurodevelopmental toxicity in zebrafish. Ecotoxicol. Environ. Saf..

[266] Yu T., Xu X., Mao H., Han X., Liu Y., Zhang H., Lai J., Gu J., Xia M., Hu C. (2022). Fenpropathrin exposure induces neurotoxicity in zebrafish embryos. Fish Physiol. Biochem..

[267] Wu Y., Wang J., Xia Y., Tang K., Xu J., Wang A., Hu S., Wen L., Wang B., Yao W. (2023). Toxic effects of isofenphos-methyl on zebrafish embryonic development. Ecotoxicol. Environ. Saf..

[268] Eghan K., Lee S., Yoo D., Kim C.H., Kim W.K. (2023). Adverse effects of bifenthrin exposure on neurobehavior and neurodevelopment in a zebrafish embryo/larvae model. Chemosphere.

[269] Yang Q., Deng P., Xing D., Liu H., Shi F., Hu L., Zou X., Nie H., Zuo J., Zhuang Z. (2023). Developmental neurotoxicity of difenoconazole in zebrafish embryos. Toxics.

[270] Yu H., Zhang J., Chen Y., Chen J., Qiu Y., Zhao Y., Li H., Xia S., Chen S., Zhu J. (2022). The adverse effects of fluxapyroxad on the neurodevelopment of zebrafish embryos. Chemosphere.

[271] Shi Q., Yang H., Chen Y., Zheng N., Li X., Wang X., Ding W., Zhang B. (2023). Developmental neurotoxicity of trichlorfon in zebrafish larvae. Int. J. Mol. Sci..

[272] Xiong Y., Wang C., Dong M., Li M., Hu C., Xu X. (2023). Chlorphoxim induces neurotoxicity in zebrafish embryo through activation of oxidative stress. Environ. Toxicol..

[273] Zhu J., Huang M., Liu C., Wang J., Zou L., Yang F., Zhu R. (2023). Curcumin protects against fenvalerate-induced neurotoxicity in zebrafish (*Danio rerio*) larvae through inhibition of oxidative stress. Ecotoxicol. Environ. Saf..

[274] Gu J., Guo L., Zhu Y., Qian L., Shi L., Zhang H., Ji G. (2023). Neurodevelopmental toxicity of emamectin benzoate to the early life stage of zebrafish larvae (*Danio rerio*). Int. J. Mol. Sci..

[275] Liu J., Xu Y., Liao G., Tu H., Huang Y., Peng T., Chen X., Huang Z., Zhang Y., Meng X. (2022). The role of ambra1 in pb-induced developmental neurotoxicity in zebrafish. Biochem. Biophys. Res. Commun..

[276] Wang Z., Li K., Xu Y., Song Z., Lan X., Pan C., Zhang S., Foulkes N.S., Zhao H. (2023). Ferroptosis contributes to nickel-induced developmental neurotoxicity in zebrafish. Sci. Total Environ..

[277] Zhao G., Hu J., Gao M., Zhu Y., Hong Y. (2022). Excessive selenium affects neural development and locomotor behavior of zebrafish embryos. Ecotoxicol. Environ. Saf..

[278] Yang X., Wang C., Yang L., Zheng Q., Liu Q., Wawryk N.J.P., Li X.F. (2022). Neurotoxicity and transcriptome changes in embryonic zebrafish induced by halobenzoquinone exposure. J. Environ. Sci..

[279] Liu S., Qiu W., Li R., Chen B., Wu X., Magnuson J.T., Xu B., Luo S., Xu E.G., Zheng C. (2023). Perfluorononanoic acid induces neurotoxicity via synaptogenesis signaling in zebrafish. Environ. Sci. Technol..

[280] Mahapatra A., Gupta P., Suman A., Ray S.S., Malafaia G., Singh R.K. (2023). Unraveling the mechanisms of perfluorooctanesulfonic acid-induced dopaminergic neurotoxicity and microglial activation in developing zebrafish. Sci. Total Environ..

[281] Chen Y., Xiao L., Gao G., He L., Zhao K., Shang X., Liu C. (2022). 2, 5-dichloro-1, 4-benuinone exposure to zebrafish embryos/larvae causes neurodevelopmental toxicity. Ecotoxicol. Environ. Saf..

[282] Torres-Ruiz M., de Alba González M., Morales M., Martin-Folgar R., González M.C., Cañas-Portilla A.I., De la Vieja A. (2023). Neurotoxicity and Endocrine Disruption Caused by Polystyrene Nanoparticles in Zebrafish Embryo. Sci. Total Environ..

[283] Xiang C., Chen H., Liu X., Dang Y., Li X., Yu Y., Li B., Li X., Sun Y., Ding P. (2023). Uv-aged microplastics induces neurotoxicity by affecting the neurotransmission in larval zebrafish. Chemosphere.

[284] Shi Q., Yang H., Zheng Y., Zheng N., Lei L., Li X., Ding W. (2023). Neurotoxicity of an emerging organophosphorus flame retardant, resorcinol bis(diphenyl phosphate), in zebrafish larvae. Chemosphere.

[285] Yang L., Zhu B., Zhou S., Zhao M., Li R., Zhou Y., Shi X., Han J., Zhang W., Zhou B. (2023). Mitochondrial dysfunction was involved in decabromodiphenyl ethane-induced glucolipid metabolism disorders and neurotoxicity in zebrafish larvae. Environ. Sci. Technol..

[286] Zhou J., Zhao Y., Dai J., Zhang K. (2023). Environmentally relevant concentrations of antidepressant mirtazapine impair the neurodevelopment of zebrafish (*Danio rerio*). Ecotoxicol. Environ. Saf..

[287] Kim J., Bang J., Ryu B., Kim C.Y., Park J.H. (2023). Flubendazole exposure disrupts neural development and function of zebrafish embryos (*Danio rerio*). Sci. Total Environ..

[288] Chen S., Wang X., Ye X., Qin Y., Wang H., Liang Z., Zhu L., Zhou L., Martyniuk C.J., Yan B. (2023). Dopaminergic and serotoninergic neurotoxicity of lanthanide phosphate (tbpo(4)) in developing zebrafish. Chemosphere.

[289] Gayathri M., Sutha J., Mohanthi S., Ramesh M., Poopal R.K. (2023). Ecotoxicological evaluation of the uv-filter octocrylene (oc) in embryonic zebrafish (*Danio rerio*): Developmental, biochemical and cellular biomarkers. Comp. Biochem. Physiol. C Toxicol. Pharmacol..

